# Trends in oligomannosylation and α1,2-mannosidase expression in human cancers

**DOI:** 10.18632/oncotarget.28064

**Published:** 2021-10-12

**Authors:** Sayantani Chatterjee, Julian Ugonotti, Ling Y. Lee, Arun Everest-Dass, Rebeca Kawahara, Morten Thaysen-Andersen

**Affiliations:** ^1^Department of Molecular Sciences, Macquarie University, Sydney, Australia; ^2^Institute for Glycomics, Griffith University, Gold Coast, Australia; ^3^Biomolecular Discovery Research Centre (BDRC), Macquarie University, Sydney, Australia; ^*^Joint senior authors

**Keywords:** oligomannose, α1,2-mannosidase, cancer, glycomics, mass spectrometry

## Abstract

Aberrant protein glycosylation is a prominent cancer feature. While many tumour-associated glycoepitopes have been reported, advances in glycoanalytics continue to uncover new associations between glycosylation and cancer. Guided by a comprehensive literature survey suggesting that oligomannosylation (Man_5–9_ GlcNAc_2_) is a widespread and often regulated glycosignature in human cancers, we here revisit a valuable compilation of nearly 500 porous graphitized carbon LC-MS/MS *N*-glycomics datasets acquired across 11 human cancer types to systematically test for oligomannose-cancer associations. Firstly, the quantitative glycomics data obtained across 34 cancerous cell lines demonstrated that oligomannosylation is a pan-cancer feature spanning in a wide abundance range. In keeping with literature, our quantitative glycomics data of tumour and matching control tissues and new MALDI-MS imaging data of tissue microarrays showed a strong cancer-associated elevation of oligomannosylation in both basal cell (*p* = 1.78 × 10^–12^) and squamous cell (*p* = 1.23 × 10^–11^) skin cancer and colorectal cancer (*p* = 8.0 × 10^–4^). The glycomics data also indicated that some cancer types including gastric and liver cancer exhibit unchanged or reduced oligomannose levels, observations also supported by literature and MALDI-MS imaging data. Finally, expression data from public cancer repositories indicated that several α1,2-mannosidases are regulated in tumour tissues suggesting that these glycan-processing enzymes may contribute to the cancer-associated modulation of oligomannosylation. This omics-centric study has compiled robust glycomics and enzyme expression data revealing interesting molecular trends that open avenues to better understand the role of oligomannosylation in human cancers.

## INTRODUCTION

Protein glycosylation, the addition of complex carbohydrates (glycans) to polypeptides, is a ubiquitous and energy-demanding post-translational modification important for a plethora of inter- and intracellular processes [1–5].

**Figure 1:**
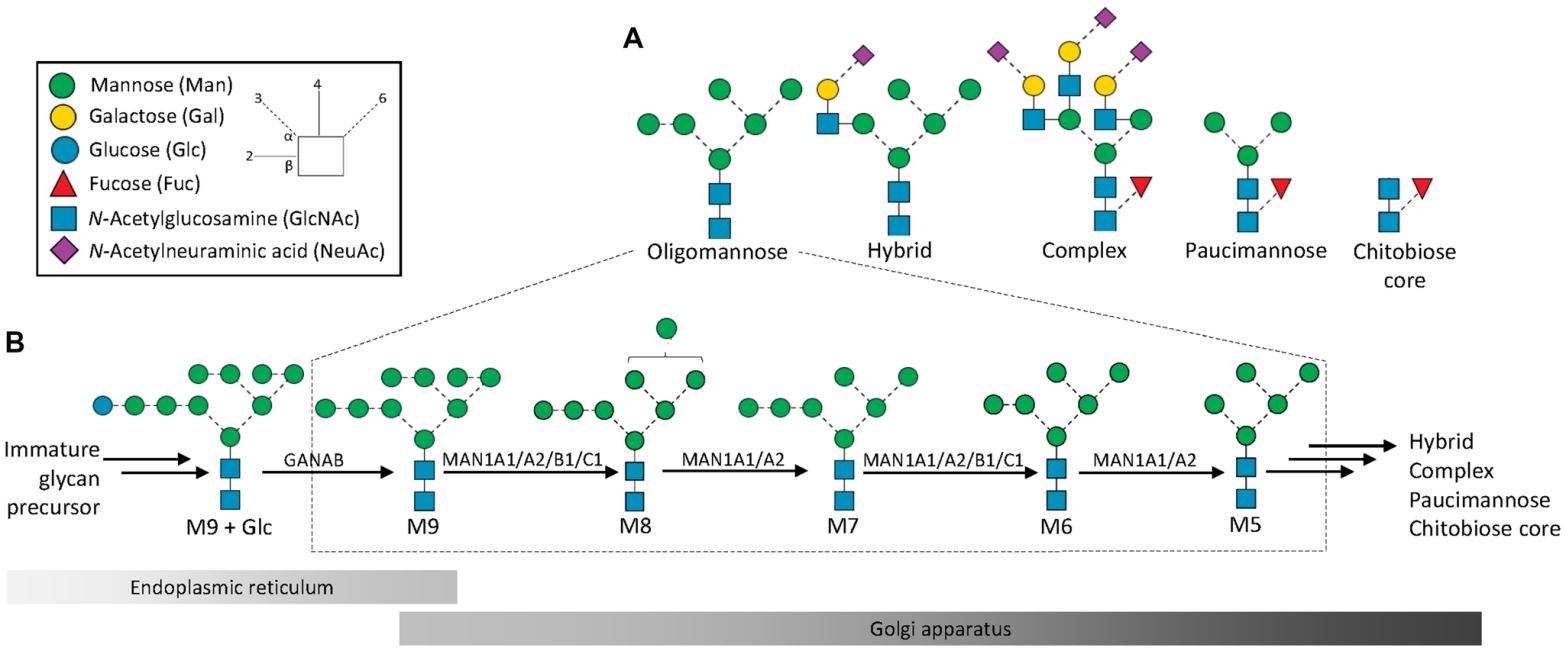
Structures, nomenclature and biosynthesis of protein oligomannosylation. (**A**) Oligomannose, one of five *N*-glycan types in human glycobiology, remains poorly studied in cancer research despite forming a large and vital part of the *N*-glycome. (**B**) Oligomannosidic *N*-glycans are formed via sequential enzymatic processing early in the biosynthetic pathway from immature Glc-capped glycan precursors in the endoplasmic reticulum (ER). The GANAB-mediated trimming of M9+Glc to M9 is followed by the processing by multiple ER- and Golgi-resident α1,2-mannosidases (MAN1A1, MAN1A2, MAN1B1, and MAN1C1) that successively trim M9 to M5. Each of the oligomannosidic glycans is known to exist as multiple isomers; only the most common isomeric form is depicted in this figure, see Supplementary Data 1 and Figure 3C for complete overview of observed isomers. The glycan processing may in cancer cells and in other cell types terminate during the oligomannose trimming reactions or continue to form more processed *N*-glycan types generating an extensive micro-heterogeneity typically observed for glycoproteins. Insert: key to monosaccharide symbols and glycosidic linkages [1].

Asparagine (Asn, *N*)-linked glycans display remarkable molecular heterogeneity despite being assembled from only few different monosaccharide building blocks including mannose (Man), galactose (Gal), fucose (Fuc), glucose (Glc), *N*-acetylglucosamine (GlcNAc) and *N*-acetylneuraminic acid (NeuAc) [5]. The template-less *N*-glycan biosynthesis begins in the endoplasmic reticulum (ER) by the transfer of immature Glc-capped glycan precursors to Asn residues located in consensus sequences of acceptor polypeptides and continues in the Golgi compartments. The biosynthesis involves successive glycan processing steps catalyzed by dozens of glycosyltransferases and glycoside hydrolases [6–8]. The resulting *N*-glycans that inherently display an extensive molecular heterogeneity are commonly classified as oligomannosidic-, hybrid- or complex-type *N*-glycans [9–12], Figure 1A. Recently, truncated *N*-glycans not fitting into these three classes were reported from various human biospecimens including the inflammation- and cancer-associated paucimannosidic- (Man_1-3_GlcNAc_2_Fuc_0-1_) and the even shorter chitobiose core- (GlcNAc_1-2_Fuc_0-1_) type *N*-glycans [12–16]. Several glycan species fit into each glycan type; for example, the M5-M9 glycans (Man_5-9_GlcNAc_2_), the focus of this study, belong to the oligomannosidic-type *N*-glycans.

Oligomannosidic *N*-glycans are synthesized early in the biosynthetic pathway, Figure 1B. Several ER- and Golgi-resident α1,2-mannosidases catalyze the successive M9-to-M5 glycan processing. Specifically, mannosyl-oligosaccharide 1,2-α-mannosidase 1A (MAN1A1, UniProtKB, P33908), mannosyl-oligosaccharide 1,2-α-mannosidase IB (MAN1A2, O60476), endoplasmic reticulum mannosyl-oligosaccharide 1,2-α-mannosidase (MAN1B1, Q9UKM7) and mannosyl-oligosaccharide 1,2-α-mannosidase 1C (MAN1C1, Q9NR34) are known to trim the outer α1,2-Man residues of nascent glycoproteins to eventually form M5 devoid of any α1,2-Man residues [2, 17]. While these first processing steps play recognized roles in glycoprotein folding, maturation and trafficking, oligomannosylation is also involved in a host of less studied extracellular functions including cell-cell and cell-extracellular matrix communication relevant for cancer and other immune-related processes including inflammation and pathogen infection [2, 18–21]. For example, reports have indicated the involvement of oligomannosylation in inflammatory processes caused by *Mycobacterium tuberculosis* [22, 23], urinary tract infections caused by *Escherichia coli* [24] and in autoimmune disorders such as systemic lupus erythromatosus [25].

Decades of intense research efforts have provided robust evidence supporting that altered protein glycosylation is an inherent feature of malignant transformation and other cancer traits, and have unearthed many glycan structures and glycoepitopes that play important roles in cancer [26]. Excellent reviews have summarized our current knowledge of the aberrant protein glycosylation underpinning cancer [26–31]. Altered expression patterns of fucosylation e.g., core and Lewis^X^ [32, 33], sialylation e.g., α2,3- and α2,6-NeuAc [34, 35] and glycan antennary branching e.g., bisecting β1,4-GlcNAc [36, 37] are prominent examples of glycan features repeatedly associated with cancer.

Despite forming a considerable part of the *N*-glycome, the mannose-terminating *N*-glycans comprising both the oligomannosidic- and paucimannosidic-type have received comparably less attention in cancer research [21]. We recently used state-of-the-art glycomics to systematically document that paucimannosylation is an overlooked feature in many cancer types including in brain (glioblastoma and neuroblastoma), blood (acute lymphocytic leukemia, ALL; acute monocytic leukemia, AML; acute promyelocytic leukemia, APL; chronic lymphocytic leukemia, CLL), bladder (BlaCa), melanoma and non-melanoma (basal cell carcinoma, BCC and squamous cell carcinoma, SCC) skin, breast (BC), hepatocellular carcinoma (HCC, liver), lung, ovarian (OvC), gastric (GC), colorectal (CRC) and prostate cancer (PCa) [38]. These findings were supported by quantitative glycomics data from nearly 500 porous graphitized carbon (PGC) LC-MS/MS datasets acquired over 10 years across laboratories around the world from a variety of cancer specimens including cultured human cancer cells and tissues from cancer patients and matching controls using a uniform analytical platform. The paucimannosidic-centric study, however, did not explore the wealth of other valuable information, including the oligomannosidic signatures, found in this compilation of content-rich *N*-glycomics datasets.

Guided by an initial literature survey suggesting that associations exist between oligomannosylation and human cancers, we have herein reinterrogated our unique collection of *N*-glycomics datasets to systematically test for oligomannose-cancer relationships across a variety of human cancer types. It transpires from this meta-analysis and supporting evidence from newly acquired matrix-assisted laser desorption/ionization mass spectrometry imaging (MALDI-MSI) and lectin flow cytometry data and enzyme expression data retrieved from well-curated cancer repositories that oligomannosylation and α1,2-mannosidase expression are significantly modulated in many types of human cancers.

## RESULTS AND DISCUSSION

### Literature suggests oligomannose-cancer associations

Despite forming a large and vital part of the human *N*-glycome, the role(s) of oligomannosylation in cancer remains poorly studied. To this end, we firstly performed a comprehensive and unbiased survey of the original research literature published over the past two decades in the field of glyco-oncology to test for a possible link between oligomannosylation and human cancers. Interestingly, we identified a considerable body of research papers that consistently reported on oligomannose elevation across 10 cancer types including bladder (BlaCa) [39], breast (BC) [40], cholangiocarcinoma (CC) [41], colorectal (CRC) [36], endometrial [42], liposarcoma [43], lung [44], ovarian (OvC) [45], oral cancer (OC) [46], and prostate cancer (PCa) [47], Table 1. These scattered but consistent observations reported by various research groups around the world were made from a diverse set of cancer specimens (cell lines, tissues and bodily fluids) using different analytical techniques (MS, HPLC, and lectin arrays). Importantly, the cancer-associated elevation of oligomannose was supported by multiple independent studies for several cancer types. Notably, we also found papers describing unchanged or reduced levels of oligomannosylation in five other cancer types including gastric (GC) [48], hepatocellular carcinoma (HCC) [49], kidney (KC) [50], pancreas (PanCa) [51], and thyroid cancer (TC) [52]. Thus, our initial literature survey indicated that interesting associations exist between oligomannosidic *N*-glycans and a subset of human cancers.

**Table 1:** Literature survey exploring a possible link between oligomannose and human cancers

Cancer type	Biological samples (*n*)	Oligomannose levels	Analytical method(s)^*^ i) analyte, ii) method	Ref
**Literature indicating oligomannose elevation in human cancers**				
Bladder cancer (BlaCa)	Five BlaCa cell lines: a) KK47, YTS1, J82, T24 (cancer) b) HCV29 (non-cancer)	M5–M9 were elevated in BlaCa cell lines	i) Amidated *N*-glycans ii) MALDI-TOF/TOF-MS	[39]
Breast cancer (BC)	a) Eight BC cell lines: SK-BR-3, MCF-7, MDA-MB-231, T-47D, ZR-75-30, Bcap37, Hs-578T (cancer) and MCF-10A (non-cancer) b) Paired BC patient tumour vs adjacent non-tumour tissues (100)	M5–M9 were elevated, particularly M8 level, in both BC cell lines and tumour tissues	i) Neuraminidase-treated APTS-labelled *N*-glycans ii) ABI-3130 sequencer (Single capillary DNA sequencer)	[53]
	Sera from: a) BC patients (7) b) healthy donors (5)	M5–M9 were elevated in sera from BC patients	i) Non-derivatized *N*-glycans ii) MALDI FT-ICR-MS and HPLC-Chip-TOF-MS	[40]
	a) Five BC cell lines: MCF-7, BT-474, SKBR-3, MDA-MB-231 (cancer) and MCF-10A (non-cancer) b) Paired BC patient tumour vs adjacent non-tumour tissues (86)	M5–M9 were elevated in both BC cell lines and tumour tissues	i) 2-AB-labeled *N*-glycans ii) PGC-LC-ESI-CID-MS/MS	[54]
	Seven BC cell lines: a) MDA-MB-435, MDA-MB-231, 578T, BT-549, NCI/ADR-RES, T-47D (cancer) b) MCF-10A (non-cancer)	M5–M9 were elevated, particularly M5–M6 levels, in both invasive and non-invasive BC cell lines	i) Permethylated *N*-glycans ii) MALDI-TOF/TOF-MS	[55]
Cholangiocarcinoma (CC, bile duct cancer)	Four CC cell lines: a) KKU2-213AL5/BL5 (metastatic) b) KKU-213A/B (parental)	M5–M9 were elevated in metastatic CC cell lines	i) Non-derivatized *N*-glycans and *N*-glycopeptides ii) nano-LC-ESI-QTOF-CID-MS/MS and RP-LC QE Plus-Orbitrap-HCD-MS/MS	[41]
	Sera from: a) CC patients (8) b) healthy donors (4)	M6 was elevated (M7-M9 reduced) in sera from CC patients	i) Permethylated *N*-glycans ii) Infusion on LTQ-ESI-CID-MS/MS	[56]
Colorectal cancer (CRC)	Paired FFPE tissue from CRC patient tumour vs adjacent non-tumour tissues (16)	M5–M9 were elevated in CRC tumour tissues	i) Amidated *N*-glycans ii) MALDI-TOF/TOF-MS	[57]
	Unpaired FFPE tissues from: a) CRC patients (stages I-IV, 18) b) healthy donors (5)	M5–M9 were elevated in advanced (stage III-IV) CRC tumour tissues	i) Non-derivatized *N*-glycans ii) MALDI-TOF/TOF-MS	[58]
	Paired CRC patient tumour vs adjacent non-tumour tissues (43)	M5–M9 were elevated in CRC tumour tissues	i) Permethylated *N*-glycans ii) PGC-LC-ESI-CID-MS/MS	[59]
	a) Unpaired tissues from: CRC patients (stages II-III, 15) and healthy donors (15) b) Unpaired sera from: CRC patients (11) and healthy donors (11)	M5–M9 were elevated, particularly M7 level, in advanced CRC tumour tissues	i) Permethylated *N*-glycans ii) MALDI-TOF-MS and RP-LC-ESI-CID-MS/MS	[60]
Endometrial cancer (uterine cancer)	a) Paired FFPE tissues from patients (6) b) Unpaired TMA from patients with (8) and without (20) lymph node metastasis	M6–M9 were elevated in tumour tissues (similar M5 distribution in tumour and non-tumour regions)	i) Non-derivatized *N*-glycans ii) MALDI-TOF/TOF-MS and PGC-LC-ESI-CID-MS/MS	[42]
Liposarcoma (fat cancer)	a) Paired FFPE tissues from patients (32) b) Unpaired TMA from patients (141) and healthy donors (6)	M6–M8 were elevated in tumour tissues	i) Non-derivatized *N*-glycans ii) MALDI-TOF/TOF-MS	[43]
Lung cancer	Paired patient tumour vs adjacent non-tumour tissues (42)	M5–M9 were elevated in tumour tissues	i) Non-derivatized *N*-glycans ii) Nano HPLC–Chip–TOF–MS	[44]
	Unpaired FFPE tissues from: a) patients with peritumoral (24) and advanced tumour (29) b) healthy donors (14)	M5–M9 were elevated in tumour tissues	i) Permethylated *N*-glycans ii) MALDI-TOF/TOF-MS	[61]
	Sera from: a) patients (64) b) healthy donors (12)	M5–M9 were elevated in sera from patients	i) Fluorescently labelled *N*-glycans ii) Lectin microarray analysis	[62]
Ovarian cancer (OvC)	Unpaired tissue samples from: a) OvC patient (83) b) healthy donors (23)	M5–M9 were elevated in OvC tumour tissues	i) Non-derivatized *N*-glycopeptides ii) QE Orbitrap-HCD-MS/MS	[63]
	FFPE OvC tissues from advanced serous OvC patients (3)	M5–M9 were elevated in OvC tumour tissues	i) Non-derivatized *N*-glycans were detected using ii) MALDI-TOF/TOF-MS	[64]
	Unpaired FFPE OvC tissues and TMA from: a) early stage (3) b) late stage (3)	M5–M9 were elevated in advanced OvC tumour tissues	i) Non-derivatized *N*-glycans ii) MALDI-TOF/TOF-MS and PGC-LC-ESI-CID-MS/MS	[45]
Oral cancer (OC)	a) Three OC cell lines: OC2 (cancer), HOK and SG (non-cancer) b) Unpaired FFPE tissues from patients (80) and healthy donors (8)	M5–M9 were elevated in both cancerous cell lines and tumour tissues	i) Permethylated *N*-glycans ii) MALDI-TOF-MS	[46]
Prostate cancer (PCa)	Sera from PCa patients with different stages: a) indolent (41) b) significant (32) c) aggressive (44)	M5–M9 were elevated, particularly, M8 level, in sera from advanced-stage PCa patients	i) 2-AB-labelled *N*-glycans ii) HILIC-UPLC with fluorescence detector	[47]
	De-identified PCa FFPE tissue block	M5–M9 were elevated in PCa tumour region	i) Non-derivatized *N*-glycans ii) MALDI FT-ICR-MS	[65]
	Sera from: a) PCa patients with grade 3 (84) and grade 4/5 (204) b) healthy donors (135)	M9 was elevated in advanced stages of sera from PCa patients (M5–M8 were not analyzed)	i) Anti-oligomannose antibodies ii) Carbohydrate microarrays and antigen-specific ELISA assays	[66]
**Literature indicating reduced/unchanged oligomannose levels in cancers**				
Gastric cancer (GC)	Sera from: a) GC patients (54) b) healthy donors (18)	Decreased M5–M9 in sera from GC patients	i) Non-derivatized *N*-glycans ii) MALDI FT-ICR-MS	[48]
Hepatocellular carcinoma (HCC, liver cancer)	Paired HCC patient tumour vs adjacent non-tumour tissues (88)	Decreased M8 in HCC tissues (M5–M7 were not analyzed, and M9 co-eluted with another analyte)	i) Neuraminidase-treated APTS-labelled *N*-glycans ii) ABI-3130 sequencer (single capillary DNA sequencer)	[49]
	Paired FFPE HCC tissues from patients (2)	Similar distribution of M8 in both HCC patient tumour and adjacent non-tumour tissues (M5–M7 and M9 were analyzed but not reported)	i) Ethyl esterified *N*-glycans ii) MALDI FT-ICR-MS	[67]
	Paired FFPE TMA from HCC patient tumour vs adjacent non-tumour tissues (16)	Decreased M8 in HCC tissues (M5–M7 and M9 were analyzed but not reported)	i) Non-derivatized *N*-glycans ii) MALDI FT-ICR-MS	[65]
	a) Paired whole HCC and normal tissue (1) b) Unpaired FFPE TMA from HCC patient tumours (with history of Hepatitis B virus, 40), liver cirrhosis (22) and non-tumour tissues (5)	Similar distribution of M5–M9 in non-tumour, cirrhotic and HCC tissues	i) Non-derivatized *N*-glycans ii) MALDI FT-ICR-MS	[68]
Kidney cancer (KC)	Paired FFPE TMA from KC stages I-IV patient tumour vs adjacent non-tumour tissues (~150)	M5–M9 mainly detected in non-tumour regions, but also present in tumour regions	i) Non-derivatized *N*-glycans ii) MALDI FT-ICR-MS and MALDI-TOF/TOF-MS	[50]
Pancreas cancer (PanCa)	Paired FFPE TMA PanCa patient tumour vs adjacent non-tumour tissues (53)	M5–M9 mainly detected in non-tumour regions, but also present in tumour regions	i) Amidated *N*-glycans ii) MALDI FT-ICR-MS and MALDI-QTOF-MS	[51]
	De-identified PanCa FFPE tissue block	M5–M9 mainly detected in non-tumour regions, but M8 is elevated in tumour regions	i) Non-derivatized *N*-glycans ii) MALDI FT-ICR-MS	[65]
Thyroid cancer (TC)	Paired TC patient tumour vs adjacent non-tumour tissues (23)	Decreased M5–M9 in TC tumour tissues	i) Ethyl esterified *N*-glycans ii) MALDI-TOF/TOF-MS	[52]

^*^All *N*-glycans were liberated from their carrier proteins using peptide:*N*-glycosidase F. Abbreviations: 2-AB: 2-aminobenzoic acid; APTS: 8-amino-1,3,6-pyrenetrisulfonic acid; FFPE: formalin-fixed paraffin-embedded; TMA: tissue microarray.

### Reinterrogation of pan-cancer LC-MS/MS glycomics datasets

Guided by the literature survey, we then revisited our collection of 467 PGC-LC-MS/MS glycomics datasets previously compiled for a recent pan-cancer study [38] to systematically test for associations between oligomannosylation and cancer. Our quantitative glycome datasets covered 11 types of human cancers including brain (glio- and neuroblastoma), BlaCa, blood (APL, AML, ALL), skin (melanoma and non-melanoma, BCC and SCC), BC, lung, HCC, GC, CRC, PCa and OvC spanning both an extensive set of human cell lines and cohorts of paired and unpaired tumour/non-tumour tissue samples. Importantly, all *N*-glycome datasets were acquired using the same analytical technique enabling not only relative *N*-glycan quantitation within each sample, but also accurate comparisons within and between sample cohorts.

The five common oligomannosidic *N*-glycans, M5-M9 (depicted in Figure 1), were consistently identified and quantified across all samples. As expected from the biosynthetic trimming process, isomers of the oligomannosidic *N*-glycans were identified across most samples. The less common M4, M8 + Glc and M9 + Glc were observed at low abundance in some samples and left out of the quantitation of the oligomannosidic glycan series. The spectral evidence for the reported structures can be found in Supplementary Data 1 and all *N*-glycome profile data are available in Supplementary Data 2 and 3.

### Cell line glycoprofiling demonstrates that oligomannose is a cancer-wide but highly variable signature

**Figure 2:**
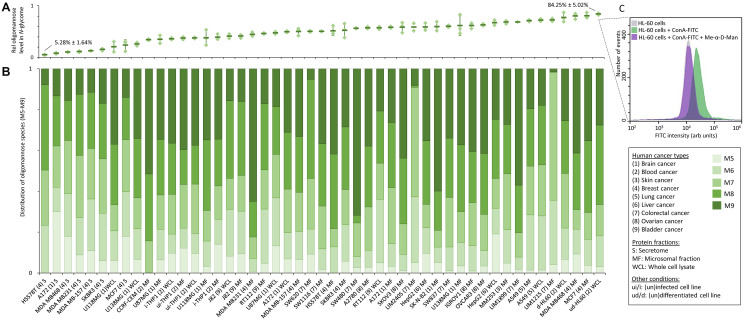
Oligomannose content across human cancer cell lines. (**A**) Total oligomannose level and (**B**) the relative distribution of individual oligomannosidic species (M5–M9) of the *N*-glycome from different protein fractions (secretome, microsomal fractions, whole cell lysates) of all investigated 34 human cancer cell lines spanning nine cancer types. See Supplementary Tables 1 and 2 for details of the studied cell lines. For A and B, data were either plotted as the mean ± SD (for *n* ≥ 3, technical replicates) or as the mean (for *n* = 2, technical duplicates). See Supplementary Data 2 and Supplementary Tables 6 and 7 for raw and tabulated quantitative glycome data, respectively. (**C**) Cell surface expression of oligomannosidic epitopes of the oligomannose-rich HL-60 cell line was shown by a surface reactivity of the mannose-recognizing concanavalin A (ConA, FITC-conjugated, green trace) using lectin flow cytometry. The ConA reactivity could be competitively inhibited by methyl-α-D-mannopyranoside (Me-α-D-Man) (purple trace) bringing the signal to background HL-60 levels as established without ConA-FITC treatment (grey trace).

Our glycomics data of 34 human cell lines spanning 9 cancer types showed that oligomannosidic *N*-glycans are universally expressed in all cancerous cells, but span a wide abundance range, Supplementary Tables 1 and 2. While the total oligomannose levels varied considerably across the investigated cancer types and between the protein fractions (5.3–84.3% of the *N*-glycome), the whole cell lysate and microsomal fractions were consistently rich in oligomannose (>20%), Figure 2A and Supplementary Table 6. In line with our previous observation [69], less oligomannosylation was consistently found in the secretome of cultured cancer cells relative to the microsomal and whole cell lysate fractions indicating that oligomannosidic *N*-glycans are mainly features of the cellular component of cancer cells. The M5-M9 glycans were all common but highly variable oligomannosidic species expressed by the cancer cell lines, Figure 2B and Supplementary Table 7. While these observations point to a significant glycan under-processing in cancer cells, incompletely processed glycoproteins still trafficking the ER and *cis*-Golgi of the secretory pathway may also contribute to the high levels of oligomannosylation detection in cancer cells. Glycan processing may be impacted by several cellular factors e.g., rate of protein synthesis, cellular growth rate (doubling time), protein trafficking time, and the levels of multiple glycan-processing enzymes and nucleotide sugars [70] as well as various protein factors [71]. While the reduced expression of several α1,2-mannosidases catalyzing the M9-to-M5 trimming process has been reported in cancer (discussed below) [72–74], the possible contribution of these many other cellular and protein factors to the oligomannose-rich glycophenotypes of cancer cells remains unexplored. Despite their relatively low secretion rate from cancers cells, oligomannosidic *N*-glycoproteins have repeatedly been found to be elevated in blood of cancer patients including those suffering from BC, CC and lung cancers [40, 56, 62] relative to the low levels of oligomannose found in the blood of healthy individuals [75, 76].

Comparative glycoprofiling of a few “normal” (non-tumourigenic) immortalized cell lines derived from breast (HMEC) and ovarian (HOSE 6.3 and HOSE 17.1) tissues and their matching cancerous cell lines from the same tissue origins including Hs-578T, MCF-7, MDA-MB-157/231/468 and SK-BR-3 breast cancer cell lines and A2780, IGROV1 and SKOV3 ovarian cancer cell lines indicated an elevation of oligomannose in the cancer cell lines (data not shown), a relationship explored in greater details below using glycomics of paired tumour/non-tumour tissue samples.

Cell surface glycoepitopes are known to play important roles in cell-cell and cell-extracellular matrix communication [1, 27, 77]. To this end, we explored the cell surface expression of oligomannosidic epitopes of the oligomannose-rich promyelocytic HL-60 cell line using lectin flow cytometry. Significant surface reactivity to the mannose-recognizing lectin, concanavalin A (ConA, FITC-conjugated) was observed, Figure 2C (green trace). Importantly, the ConA reactivity could be competitively inhibited by methyl-α-D-mannopyranoside (Me-α-D-Man) (purple trace) bringing the signal back to the base levels established for HL-60 without ConA-FITC treatment (grey trace). The putative cell surface expression of oligomannosidic epitopes on HL-60 cells and possibly other cancer cells is interesting as it suggests roles of oligomannose in cancer cell communication and may open for novel glycan-centric diagnostic approaches as well as new treatment strategies such as chimeric antigen receptor T-cell therapy directed to surface-exposed oligomannose [78]. Supporting the cell surface expression of oligomannose, several mannose-recognizing receptors such as macrophage mannose receptor (CD206), DC-SIGN [79], langerin [80] and dectin-2 [81] have been found to recognize oligomannosidic *N*-glycans expressed on tumour cells [82]. The recent development of a comprehensive oligomannose microarray may open for the identification of additional receptors recognizing oligomannose-decorated cancer cells and tissues [83].

### Comparative tissue glycomics and MALDI-MS imaging confirm oligomannose elevation in a subset of cancer types

**Figure 3:**
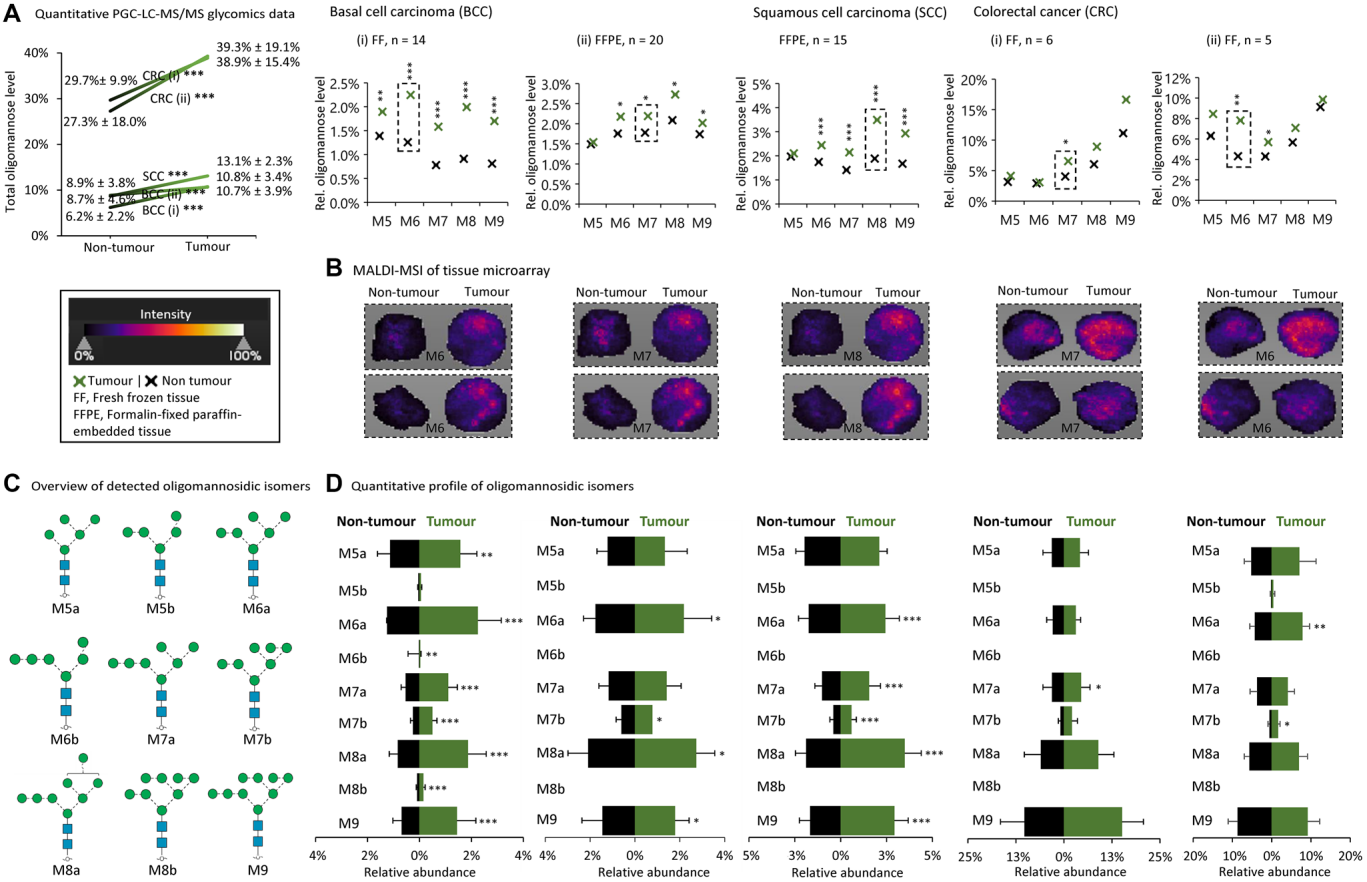
Tissue glycomics and MALDI-MSI data show oligomannose elevation in a subset of cancers. (**A**) Some cancer types displayed elevated oligomannose levels as demonstrated by quantitative glycomics of donor-paired tumour and non-tumour tissues from patients with basal cell carcinoma (BCC, i, FF; ii, FFPE tissues), squamous cell carcinoma (SCC) and colorectal cancer (CRC, i–ii, FF tissues). See Supplementary Tables 3 and 4 for details of the investigated tissues. See Supplementary Data 3 and Supplementary Table 8 and 9 for all raw and tabulated glycome data, respectively. Statistics was performed using paired and unpaired two-tailed *t-test*s (*n*, patient samples, as indicated). ^*^ *p* < 0.05, ^**^ *p* < 0.001, ^***^ *p* < 0.0001, n.s. not significant (*p* ≥ 0.05). (**B**) The MALDI-MSI data were from a TMA slide of unpaired tumour and non-tumour tissues. Representative examples of oligomannosidic *N*-glycans displaying prominent regulation in the tumour tissues have been provided (boxed, broken line). See key for MALDI-MSI intensity range and Supplementary Table 5 for details of the TMA used for the MALDI-MSI. (**C**) Overview of the oligomannosidic isomers detected from the PGC-LC-MS/MS glycomics experiments. (**D**) Quantitative profile of the detected oligomannosidic isomers from donor-paired tumour and non-tumour tissues of the same cancer types and samples explored in (A). Statistics was performed using paired two-tailed *t-test*s (n, patient samples, as indicated). ^*^ *p* < 0.05, ^**^ *p* < 0.001, ^***^ *p* < 0.0001, n.s. not significant (*p* ≥ 0.05).

Next, *N*-glycomics data from 126 paired and unpaired tumour/non-tumour tissues (fresh frozen, FF and formalin-fixed paraffin-embedded, FFPE) from various patient cohorts spanning seven cancer types were re-analyzed, Supplementary Tables 3 and 4. A subset of the investigated cancer types including non-melanoma BCC (FF, *n* *=* 14; FFPE, *n* *=* 20) and SCC (FFPE, *n* *=* 15), and CRC (FF, *n* *=* 5–6) showed strong tumour-associated elevation of oligomannose (*p* < 0.0001 for all three cancer types, paired two-tailed *t-test*s), Figure 3A. Most of the individual oligomannosidic *N*-glycan species showed a significant elevation in the tumour tissues relative to the adjacent control tissues albeit no consistent patterns of the relative M5-M9 distribution between the studied cohorts and cancer types could be identified.

MALDI-MSI of glycans, a powerful tool to spatially profile liberated glycans from biological specimens including TMAs (as used here, see Supplementary Table 5 for meta-data) [64, 84, 85], recapitulated the quantitative glycomics data by consistently showing a higher expression of specific oligomannosidic *N*-glycans in the tumour relative to non-tumourigenic tissues in BCC, SCC and CRC, Figure 3B.

The tumour tissue PGC-LC-MS/MS data revealed as expected the presence of various isomers (M5a, M5b, M6a, M6b, M7a, M7b, M8a, M8b and M9) of the oligomannosidic species, Figure 3C. The quantitative profiling of each oligomannosidic isomer provided another level of structural insight into the regulation of the oligomannosidic *N*-glycans in BCC, SCC and CRC, Figure 3D.

In keeping with the observations made from our literature survey, our quantitative glycomics and MALDI-MSI data supported that several cancer types showed unchanged or slightly reduced levels of oligomannose in tumour tissues relative to matching non-tumour tissues including HCC (FFPE, *n* = 3) and GC (FF, *n* = 3) (*p* ≥ 0.05 for all cancer types, paired two-tailed *t-test*s) and PCa (FF, *n* = 5 and 55) (*p* ≥ 0.05, unpaired two-tailed *t-test*s), Supplementary Figure 1. While late-stage CLL also did not show oligomannose elevation relative to early-stage disease (FF, *n* = 4) (*p* ≥ 0.05, unpaired two-tailed *t-test*s), all CLL tissues notably displayed very high levels of oligomannosylation (>60%). Our data therefore did not point to a progression-dependent elevation of oligomannose in CLL, but follow-up analysis of B cells from healthy donors are still required to determine if raised oligomannose is a disease characteristic of CLL.

H&EE staining confirmed that the investigated spots were indeed from tumour and non-tumour regions across the TMA, Supplementary Figure 2A. The complete set of MALDI-MSI data depicting the spatial distribution and relative abundance of all the oligomannosidic species in the tumour and non-tumour tissues investigated in this study is provided in Supplementary Figure 2B. While this study did not address the cellular origin(s) of the elevated oligomannose in the heterogeneous tumour micro-environment (discussed below), MALDI-MSI and glycoproteomics with cell annotation of ovarian [45] and prostate [65, 86] tumour tissues have previously indicated that oligomannose-rich glycophenotypes are prominent features of cancer cells as oppose to the stroma, immune cells and other cell types forming important auxiliary components of the tumour microenvironment.

Collectively, these studies therefore indicate that raised oligomannose levels originate directly from the cancer cells, however, this aspect and the still elusive protein carriers of cancer-associated oligomannose require further investigation. Site-, protein- and even tissue-specific information, which can provide important mechanistic insight into the cancer-associated oligomannose elevation, can now be obtained with emerging glycoproteomics [86, 87] and advanced cell-sorting and characterization technologies [88]. Sensitive and quantitative proteomics often performed in concert with glycoproteomics may also be able to profile the α1,2-mannosidases and other glycan-processing enzymes to inform more precisely on their regulation and hence support the link to altered oligomannosylation in cancer.

### Altered α1,2-mannosidase expression in human cancers

**Figure 4:**
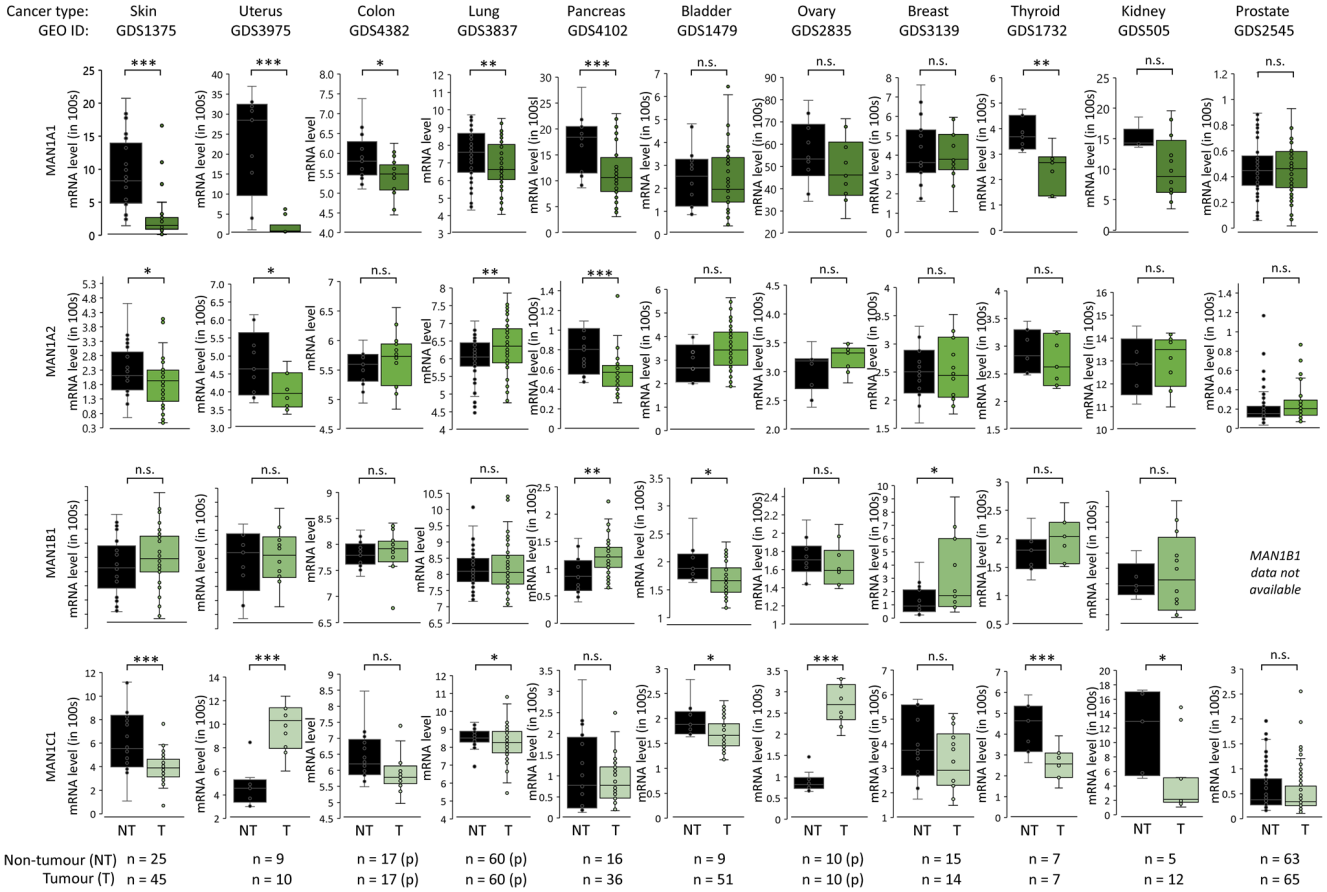
α1,2-mannosidase expression patterns in cancer. mRNA expression of MAN1A1, MAN1A2, MAN1B1 and MAN1C1 in paired (p) and unpaired tumour (different shading of green data points) and non-tumour (black data points) tissues across cancer types based on data extracted from the Gene Expression Omnibus (GEO). Statistics was performed using paired and unpaired two-tailed *t-test*s (*n*, sample numbers, as indicated below the graphs). ^*^ *p* < 0.05, ^**^ *p* < 0.001, ^***^ *p* < 0.0001, n.s. not significant (*p* ≥ 0.05).

We then investigated a possible connection between the biosynthetically relevant α1,2-mannosidases and oligomannose expression in cancer tissues. Using the Gene Expression Omnibus (GEO) database, the transcript (mRNA) expression of MAN1A1, MAN1A2, MAN1B1 and MAN1C1 measured from paired and unpaired tumour/non-tumour tissues were investigated, Figure 4 and Supplementary Data 4A. Mining the transcript data in the context of CRC and skin cancers for which our glycomics data showed a cancer-associated elevation of oligomannosylation (see Figure 3), the expression analysis suggested a significant reduction in MAN1A1 in tumour compared to non-tumour tissues (both *p* < 0.05). Further, MAN1A2 and MAN1C1 were also reduced in tumour relative to non-tumour tissues for skin cancer (both *p* < 0.05). Albeit less consistent, trends of α1,2-mannosidase down-regulation were also observed for lung, bladder, and breast cancers that based on the literature survey also showed an elevation in oligomannosylation. While some cancer types with increased oligomannosylation such as uterine and ovarian cancers showed trends towards a decrease in MAN1A1 expression in tumour compared to non-tumour tissues, MAN1C1 interestingly showed the opposite pattern by being significantly elevated (both *p* < 0.0001). Cancer types that showed no elevation in oligomannose (pancreatic, thyroid and kidney cancers) also displayed some degree of α1,2-mannosidase regulation suggesting that complex and yet-to-be-understood relationships exist between oligomannosylation and the processing enzymes in cancer.

We therefore sought to explore further the link between the α1,2-mannosidases and oligomannose expression in more simple biological systems. For this purpose, we compared transcript data of all four α1,2-mannosidases from five cancer cell lines (HL-60, CCRF-CEM, Hs-578T, SKOV3 and A549) retrieved from the Cell Miner^™^ database with our glycomics data from the same cell lines, Supplementary Figure 3A and Supplementary Data 4B. Correlation analyses showed that the oligomannose levels correlated negatively with the expression of MAN1A1 (*r* = –0.809), MAN1A2 (*r* = –0.614) and MAN1B1 (*r* = –0.966) while MAN1C1 showed a positive correlation (*r* = +0.728). These observations support the complex interplay between the α1,2-mannosidases and the oligomannose levels in cancer.

To investigate whether the reduced expression of the α1,2-mannosidases observed in some cancers may be linked to high mutation rates of these enzymes, we explored the frequency of simple somatic mutations (SSMs) of the four α1,2-mannosidases across 11 cancer types (skin, uterus, colon, lung, gastric, bladder, ovary, breast, liver, kidney and prostate) using data retrieved from The Cancer Genome Atlas (TCGA), Supplementary Figure 3B and Supplementary Data 4C. To enable comparisons across datasets, the cancer types were selected to match the cancers covered by our glycomics data and the literature survey. Interestingly, relatively high SSM rates of all four α1,2-mannosidases were found in cancer types displaying oligomannose elevation i.e., skin, uterus, colon, and lung cancers while lower α1,2-mannosidase SSM rates were observed for liver, gastric, kidney and prostate cancers not featuring elevated oligomannosylation. Further investigations are required to determine how the SSMs impact the expression and activity of the α1,2-mannosidases.

While these observations based on publicly available expression datasets collectively point to the involvement of the α1,2-mannosidases in the cancer-associated elevation of oligomannose, attempts to relate more precisely the enzyme expression to the oligomannose levels observed across the different cancer types, did not reveal a clear and consistent pattern possibly highlighting the complex interplay between the four α1,2-mannosidases and oligomannose. For this study, we investigated three well established public repositories (GEO, Cell Miner^™^ and TCGA) all packed with valuable data and resources, but other public databases including Gene Expression Atlas [89], SAGEmap [90] and XenaBrowser [91] can be explored further to generate a more complete picture of the regulation of the α1,2-mannosidases in human cancers.

Interestingly, a recent study showed that genetic disruption of the α1,2-mannosidases in HEK293 cells resulted in oligomannose elevation [92]. While the single knockouts of MAN1A1 and MAN1A2 did not exhibit changes, the double knockout HEK293 cell line of these two prominent α1,2-mannosidases displayed a significant increase in oligomannose. Additionally, an abundance of M9-M8 structures were found in the triple knockout of MAN1A1, MAN1A2 and MAN1B1. These observations carried out under controlled conditions not only confirm the involvement of the α1,2-mannosidases in oligomannose processing, but also support a negative correlation between specific α1,2-mannosidases i.e., MAN1A1, MAN1A2 and MAN1B1 and oligomannose reported here (see Supplementary Figure 3A) and indicate that these glycan-processing enzymes are in part able to compensate for each other.

MAN1A1 has repeatedly been reported to be down-regulated in a variety of metastatic cancer cell lines including in HCC [93], CC [41], OvC [94] and PCa [95] relative to their non-metastatic counterparts. Thus, it can be speculated that MAN1A1 and/or the resulting oligomannosidic *N*-glycan products may contribute (directly or indirectly) to the aberrant growth and dissemination of some cancer types. Moreover, a recent study showed that reduced MAN1A1 expression and oligomannose elevation are features of advanced-stage BC tumour tissues with high tumour aggressiveness relative to early-stage BC tumour tissues and suggested that these glyco-phenotypic characteristics lead to impaired survival in BC patients [72].

We found comparably less literature linking the other biosynthetically relevant α1,2-mannosidases to cancer. In a recent study, MAN1B1 expression was reported to be up-regulated in BlaCa relative to normal tissues and was found to be a regulator of cell proliferation [74]. Other studies have indicated that MAN1C1 is a tumour suppressor in KC [73] and HCC [96]. Finally, low expression of MAN1C1 (and a concomitant up-regulation of MAN1A1) was also reported in HCC (hepatitis B-positive) stage 1 relative to stage 2 and 3 further supporting that MAN1C1 plays roles in tumour suppression, whereas MAN1A1 was suggested to be an oncogene in hepatocarcinogenesis [96]. We did not find any literature on the role of MAN1A2 in cancer.

Collectively, our glycomics data, the existing glyco-oncology literature and enzyme expression data retrieved from public cancer repositories suggest that several α1,2-mannosidases impact oligomannose expression in complex and yet-to-be-fully understood ways in some types of human cancer.

### Limitations of this study

This study reports trends in oligomannose levels and α1,2-mannosidase expression patterns across several cancer types. The communicated trends were backed by a considerable volume of robust PGC-LC-MS/MS glycomics data and public transcriptomics data and were supported by complementary experimental data and a body of peer-reviewed literature. While these observations contribute to a better understanding of oligomannose in cancer, further exploration is required to identify the mechanistic link between oligomannosylation and cancers and several limitations of the study should be pointed out.

The study identified molecular trends from a diverse set of data obtained from various clinical sources rather than from a single uniform and controlled experiment. The presented data therefore feature a relatively high degree of variability arising, in part, from the multiple cohorts of clinical samples studied. While age and gender have previously been reported to impact glycosylation [97] and therefore may be considered potential confounding factors for the glycomics data reported herein, we did not identify any impact from age and gender possibly due to the relatively small and heterogenous sample cohorts included in this study. Other factors that may contribute to glycome variation including the exact location of the sampled tumour and non-tumour tissues, the nature and extent of the immune cell infiltrate, the metastatic potential and the disease progression of the studied tumours, as well as the ethnicity and blood types of patients and donors were not controlled for in this study. Follow up studies performed on a larger and more homogenous cohort comprising appropriate controls including age- and gender-matched healthy donors and donors exhibiting inflammation and which appropriately correct for confounding factors are required to confirm and expand on the trends reported herein.

The cultured cancer cell lines also profiled with this study represent comparably more homogenous specimens. However, given the fact that the studied cell lines arise from different tissue origins, were generated with various immortalization procedures, featured undisclosed and possibly highly variable passage numbers, were grown under diverse culture conditions and were sampled using different methods to extract different protein fractions, the glycomics data from these experiments are expected to vary considerably and may therefore contribute to the large spread in oligomannosylation levels observed across the glycoprofiling data of the cancer cell lines.

Adding to the limitations of the biological samples studied, several technical limitations should be mentioned. The H&EE staining supporting the MALDI-MSI data (performed on the same spots) was limited to a small and in some cases incomplete or disintegrated tissue core from the TMA. We were therefore unable to accurately correlate the observed spatial changes in oligomannosylation with specific tumour and non-tumour regions of the studied tissue sections. Delineating more effectively the spatial distribution of glycans in tumour tissues using MALDI-MSI, ideally performed on a higher number of replicates preferably from paired tumour and adjacent non-tumour tissues from the same patients from which glycomics data are also available, will provide a better understanding of which cells in the complex tumour microenvironment contribute to the elevation of oligomannose observed in some cancers.

For the lectin flow cytometry used to probe the cell surface oligomannosylation, concanavalin A is known to recognize not only mannosyl epitopes but also cross-react with other glycoepitopes including biantennary complex-type *N*-glycans and terminal glucose moieties [98]. While we showed that Me-α-Man competitively reduced ConA reactivity, the cell surface expression of oligomannosidic epitopes should therefore be confirmed using other mannose-recognizing lectins (ideally performed with and without endoglycosidase H treatment and α-Man competitors as appropriate controls) and/or using orthogonal methods including MS-based glycomics profiling after subcellular fractionation or cell surface capture or via lectin arrays [99] and should be studied more widely across other cancer cell lines to determine if cell surface expression of oligomannosylation is a general feature of cancer cells.

## MATERIALS AND METHODS

### Literature survey

The glycomics and glycobiology literature was comprehensively surveyed in attempts to test for a link between oligomannose and human cancer using the PubMed (https://pubmed.ncbi.nlm.nih.gov/) and Google Scholar (https://scholar.google.com.au/) search engines. Combinations of search keywords were employed including “high-mannose” (with/without hyphen), “extended mannose”, “oligomannose”, “human”, “cancer” and “carcinoma”. Other keywords including “*N*-glycan”, “glycoprofiling”, and “*N*-glycome profiling” were used to retrieve additional literature. All types of human cancers, biological samples, and experimental and analytical methods were considered in the literature search. Only original contemporary research papers published from 2000-2021 were considered. Key details and the main findings from these papers were included in Table 1 without any new or additional data reinterrogation. Relevant reviews and associated research papers were also surveyed to ensure that the existing literature was exhaustively covered by our search efforts.

### Reinterrogation of cancer *N*-glycomics datasets

The biological samples, sample handling and data acquisition methods used to generate the compilation of LC-MS/MS glycomics datasets reinterrogated with this study have been exhaustively described in Chatterjee et al., 2018 [38]. The details are also found in the Extended Experimental Methods in the Supplementary Information.

In short, the biological samples included cultured human cancerous and non-cancerous cell lines spanning 9 different cancer types including brain (glioblastoma and neuroblastoma), blood (APL, AML, ALL), melanoma, BC, lung, HCC, CRC, OvC and BlaCa, Supplementary Tables 1 and 2, and tumour and adjacent control tissue samples from six different cancer types including blood (CLL), non-melanoma skin (BCC and SCC) cancer, GC, HCC, CRC and PCa, Supplementary Tables 3 and 4.

Proteins were extracted from the biological specimens, and the *N*-glycans released and handled as previously described [100, 101]. Proteins from each cell line were extracted from either the secretome, microsomal fraction and/or whole cell lysate and spotted in technical triplicates for three separate rounds of *N*-glycan release and analysis except for the proteins extracted from the CRC cell lines that were spotted in technical duplicates. No technical replicates were performed for the tissue samples that instead were studied as biological replicates from different patients. In short, the *N*-glycans were liberated using peptide: *N*-glycosidase F and quantitatively profiled in their reduced but otherwise non-derivatized alditol form using PGC-LC-MS/MS in negative ion polarity using linear and 3D ion trap mass spectrometers.

The raw data of all 467 LC-MS/MS glycomics datasets (available via the MassIVE Consortium, ftp://massive.ucsd.edu/MSV000083727/) were for the purpose of this study reinterrogated using the ESI-compass data analysis 4.0 software v1.1 (Bruker Daltonics) or Xcalibur v2.2 (Thermo Scientific) using assisting software i.e., GlycoMod (http://www.expasy.ch/tools/glycomod) and GlycoWorkBench v2.1 and manual *de novo* glycan sequencing as previously described [102, 103]. Glycan isomers were identified based on their monoisotopic precursor mass, MS/MS fragmentation pattern and their relative and absolute PGC-LC retention time [104]. The relative abundances of the individual *N*-glycans were determined from relative area-under-the-curve measurements based on extracted ion chromatograms performed for all charge states of the precursor ions using RawMeat v2.1 (Vast Scientific), QuantAnalysis software v2.1 (Bruker) and Skyline (64-bit) v20.1.0.76 [104]. The total relative level of all oligomannosidic *N*-glycans was determined as a proportion of the entire *N*-glycome. For that purpose, only *N*-glycans observed above a limit-of-quantitation threshold of 0.01% relative abundance were considered. Further, the relative distribution of the individual oligomannosidic *N*-glycans was determined based on their relative abundance as a proportion of all observed oligomannosidic *N*-glycans. Five major oligomannose species, M5-M9, some with at least two isobaric isomers were consistently identified across the investigated samples, Supplementary Data 1. Low levels of M4, M8 + Glc and M9 + Glc not fitting the common definition of oligomannose were observed in some samples and were excluded from the quantitative profiling of oligomannose. Supplementary Data 2 (cell lines) and Supplementary Data 3 (tissues) summarize key information of all identified glycans for each cancer type including details of the structure, composition, observed and theoretical molecular mass and mass deviation, PGC-LC retention time, area-under-the-curve value and relative abundance of the oligomannosidic species.

### Lectin flow cytometry

HL-60 cells (ATCC^®^, CCL-240^™^) were cultured in Roswell Park Memorial Institute-1640 media supplemented with 10% (v/v) heat-inactivated fetal bovine serum (Sigma-Aldrich) and maintained at 37°C with 5% CO_2_. Cells were pelleted by centrifugation at 300 × g, washed twice in phosphate buffered saline (PBS), and resuspended in PBS with 1% (w/v) bovine serum albumin (BSA, Sigma-Aldrich) before cell counting and viability determination using 0.4% (w/v) trypan blue staining (Sigma-Aldrich). A total of 5 × 10^5^ cells were used for each flow cytometry experiment. Cells were pelleted, washed once in PBS/1% BSA, resuspended in 100 μl concanavalin A-FITC (ConA-FITC, 1 μg/ml, Sigma-Aldrich) in the presence or absence of 0.5 M methyl-α-D-mannopyranoside (Me-α-Man) and then incubated in the dark on ice for 30 min. Cultured HL-60 cells without ConA-FITC treatment were used as a control. After incubation, cells were washed twice with PBS/1% BSA and resuspended in 500 μl PBS/1% BSA for analysis on a CytoFLEX S flow cytometer (Beckman Coulter, Australia). The conjugated FITC was excited using a 488 nm laser and emission read at 525 nm using a 525/40 band-pass filter with a gain setting of 300. Data were acquired and stored in .fcs file format, imported into R v4.1.0 and analyzed with the R-package flowCore v2.2.0.

### Tissue microarray (TMA)-based matrix-assisted laser desorption/ionization mass spectrometry imaging (MALDI-MSI)

A TMA slide with 15 different tumour tissues from patients suffering from various types of cancer and matching normal control tissues from healthy individuals (unpaired tumour/non-tumour sets, deposited on the TMA in technical duplicates) was obtained (Biochain, USA, catalogue #T8235712-5, lot #B306118). The patient meta-data have been provided in Supplementary Table 5. The slide was pre-coated with indium tin oxide to generate a conductive surface for MALDI-MSI.

The TMA was rehydrated and processed using a standard procedure for citric acid-based antigen retrieval [105]. Briefly, the tissue sections were heated to 60°C for 1 h on a heating block, washed twice in 100% (v/v) xylene for 5 min followed by serial dilutions in ethanol (100%, 90%, 70%, 50%, v/v) for 2 min each. Sections were then washed twice in Milli-Q water for 5 min. Next, the slides were boiled in 10 mM citraconic acid (pH 3.0) for 20 min on a steamer. Finally, tissue sections were immersed twice in 10 mM ammonium bicarbonate for 1 min and dried at room temperature in a humidity chamber. Peptide-*N*-glycosidase F (PNGase F PRIME^™^; N-Zyme Scientifics, USA) was sprayed onto the tissue sections using a TM-Sprayer (HTX-Imaging, USA) at a concentration of 0.1 μg/μl, flow rate of 25 μl/min, 15 passes, criss-cross pattern, velocity of 1200 mm/min, and track spacing of 3.0 mm. The nitrogen gas pressure was maintained at 10 psi. Tissue sections were incubated at 37°C for 2 h in a humidity chamber. A homogenous matrix (α-cyano-4-hydroxycinnamic acid, 10 mg/ml in 50% (v/v) acetonitrile/0.1% (v/v) trifluoroacetic acid) was applied to tissues using the TM-sprayer. The applied settings were: 8 passes, 0.1 ml/min flow rate, 10 psi nitrogen pressure, 80°C capillary temperature, criss-cross pattern, velocity of 1200 mm/min, and track spacing of 3.0 mm.

The MSI data were acquired using a rapifleX MALDI-TOF/TOF mass spectrometer (Bruker Daltonics, Germany) controlled by flexControl (v4) and flexImaging (v5.1). Instrument settings were: *m/z* 920–3200, 5 kHz laser repetition rate and 2.5 GS/s. A total of 400 shots were acquired using a smartbeam 3D laser at 35 μm spatial resolution. The instrument was calibrated on-tissue using theoretical *m/z* values of known glycans prior to data acquisition. The MALDI-MSI data were analyzed using SCiLS Lab v2016b, SCiLS (Bruker Daltonics, Germany). Raw data were pre-processed by baseline subtraction and normalization to the total ion count as previously shown [84, 85, 105].

Following the MALDI-MSI analysis, the matrix was eluted from the slides using 70% (v/v) ethanol and tissue sections were stained using hematoxylin-eosin (H&EE) staining to validate that the investigated tissue spots were from tumorigenic and non-tumour regions across the TMA using a standard protocol as described previously [105].

### Expression data of human α1,2-mannosidases

Transcript (mRNA) expression data of MAN1A1, MAN1A2, MAN1B1 and MAN1C1 from paired and unpaired tumour and non-tumour tissues across 12 cancer types were obtained from the Gene Expression Omnibus (GEO) database (https://www.ncbi.nlm.nih.gov/geo/) (Supplementary Data 4A) [106, 107]. Projects with the following GEO accession IDs (primary site) were considered: GDS1375 (skin), GDS3975 (uterus), GDS4382 (colon), GD3837 (lung), GDS1210 (gastric), GDS1479 (bladder), GDS2835 (ovary), GDS4102 (pancreas), GDS3139 (breast), GDS505 (kidney), GDS1732 (thyroid) and GDS2545 (prostate). Only human cancer types covered by our literature survey and/or our collection of glycomics datasets were included in this analysis. Other inclusion criteria included the availability of data from both non-tumour and tumour (primary site) tissues from a minimum of five patients per cohort.

Transcript (mRNA) expression data of MAN1A1, MAN1A2, MAN1B1 and MAN1C1 from select cancer cell lines were obtained using the RNAseq NCI-60 data [108] via the CellMiner^™^ database (http://discover.nci.nih.gov/cellminer) (Supplementary Data 4B) [109]. Only cell lines from primary tumour sites and with matching glycomics data from whole cell lysates and/or microsomal fractions were included in this analysis to limit the variability among the compared samples by avoiding unwanted influence from the metastatic transformation. Thus, cancer cell lines from metastatic sites and where glycomics data only were available for the secretome were left out. Five cell lines matched these inclusion criteria i.e., HL-60 and CCRF-CEM (blood cancer), Hs-578T (BC), SKOV3 (OvC) and A549 (lung cancer). The relative oligomannose abundance and the transcript levels of the four α1,2-mannosidases were normalized to 1 across the five cell lines. Correlation between the oligomannose and α1,2-mannosidase expression levels were determined using Pearson correlation.

The rates of simple somatic mutations (SSMs) of four α1,2-mannosidases were obtained from The Cancer Genome Atlas (TCGA) (https://www.cancer.gov/tcga) (Supplementary Data 4C) [110]. Data obtained from unique cancer primary sites including 11 projects with the following accession IDs (primary site) were considered: TCGA-SKCM (skin), TCGA-UCEC (uterus), TCGA-COAD (colon), TCGA-LUSC (lung), TCGA-STAD (stomach/gastric), TCGA-BLCA (bladder), TCGA-OV (ovary), TCGA-BRCA (breast), TCGA-LIHC (liver), TCGA-KIRC (kidney) and TCGA-PRAD (prostate).

### Statistics

Statistical tests of the glycomics data and the α1,2-mannosidase expression data were performed using paired and unpaired one- or two-tailed Student’s *t-test*s after testing for normality using the Kolmogorov-Smirnov test. Significance has been consistently indicated as ^*^ *p* < 0.05), ^**^ *p* < 0.001), ^***^ *p* < 0.0001) while n.s. denotes a lack of significance (*p* ≥ 0.05). The number of patient samples (*n*) for the different tissue analyses has been indicated. Cell line glycoprofiling data were generated from at least two or three technical replicates for each experiment. Data have generally been plotted as the mean ± standard deviation (SD) where *n* ≥ 3.

## CONCLUSIONS

Guided by a comprehensive literature survey pointing to a hitherto under-explored association between oligomannosylation and human cancers, we have here reinterrogated a large volume of PGC-LC-MS/MS glycomics datasets generated from a diverse set of cancer cell lines and valuable cohorts of patient tissues. Our quantitative glycomics data and supporting qualitative MALDI-MSI data demonstrate that the oligomannose content varies greatly across different tumour micro-environments and that some cancers show a trend towards increased oligomannosylation levels. Also deserving further exploration, the lectin flow cytometry data indicated that oligomannose may in some cancers form cell surface epitopes. Finally, data from well-curated cancer repositories suggested that aberrant expression of several α1,2-mannosidases may contribute to the modulation of oligomannose in some types of cancers.

Conclusively, this omics-centric study has compiled considerable volumes of robust data and revealed interesting trends in oligomannosylation and α1,2-mannosidase expression in several cancers that collectively serve as an important resource to further explore the role of oligomannosylation in human cancers.

## SUPPLEMENTARY MATERIALS









## Abbreviations

2-AB: 2-aminobenzoic acid
ALL: acute lymphocytic leukemia
AML: acute monocytic leukemia
APL: acute promyelocytic leukemia
APTS: 8-amino-1,3,6-pyrenetrisulfonic acid
Asn: asparagine
BC: breast cancer
BCC: basal cell carcinoma
BlaCa: bladder cancer
BPH: benign prostatic hyperplasia
BSA: bovine serum albumin
CC: cholangiocarcinoma
CLL: chronic lymphocyte leukemia
ConA: concanavalin A
CRC: colorectal cancer
ER: endoplasmic reticulum
FF: fresh frozen
FFPE: formalin-fixed paraffin-embedded
Fuc: fucose
Gal: galactose
GC: gastric cancer
GEO: Gene Expression Omnibus
Glc: glucose
GlcNAc: *N*-acetylglucosamine
H&EE: hematoxylin and eosin
HCC: hepatocellular carcinoma
KC: kidney cancer
LC-MS/MS: liquid chromatography tandem mass spectrometry
MALDI-MSI: matrix-assisted laser desorption/ionization mass spectrometry imaging
Man: mannose
Me-α-Man: methyl-α-D-mannopyranoside
NeuAc: *N*-acetylneuraminic acid
OC: oral cancer
OvC: ovarian cancer
PanCa: pancreas cancer
PBS: phosphate buffered saline
PCa: prostate cancer
PGC: porous graphitized carbon
SCC: squamous cell carcinoma
SD: standard deviation
SSM: simple somatic mutation
TC: thyroid cancer
TCGA: The Cancer Genome Atlas
TMA: tissue microarray
