# Supplementary material for: Trends in oligomannosylation and α1,2-mannosidase expression in human cancers
Source: Oncotarget. 2021 Oct 12;12(21):2188–205. doi: 10.18632/oncotarget.28064 (PMC8522845; doi:10.18632/oncotarget.28064)
Supplement: Supplementary file 1 [file oncotarget-12-2188-s001.pdf]

Trends in oligomannosylation and α1,2-mannosidase expression in human cancers

SUPPLEMENTARY MATERIALS

Extended experimental methods

Human ethics

Ethics approvals were obtained from the local ethic committees relevant to the individual sample cohorts investigated in this study. Specifically, the study of i) chronic lymphocyte leukemia (CLL) tissues was approved by the human research ethics committee (HREC) at the University of Sydney, Australia (project #8935), ii) non-melanoma tissues (basal cell carcinoma, BCC and squamous cell carcinoma, SCC) was approved by ethics committee at University of Leipzig (ethics #127-11-18042011), iii) gastric tissues was approved by *Comitato Etico Regione Toscana* (Tuscany, Italy) and confirmed to be in line with the ethical guidelines of the Declaration of Helsinki (1975), iv) cirrhotic and hepatocellular carcinoma (HCC) liver tissues was approved by the ethics committee at *Klinička Bolnica Merkur* at Zagreb, Croatia, v) colorectal cancer (CRC) tissues was obtained from Sydney South West Area Health Service (ethics #X08-0614) and Department of Pathology, Yonsei University and Liver Cancer Specimen Bank of the National Research Resource Bank program of the Korea Science and Engineering Foundation of the Ministry of Science and Technology (Seoul, South Korea) (ethics #5201100040) respectively, vi) prostate cancer (PCa) and matching benign prostatic hyperplasia (BPH) tissues was approved by Faculdade de Medicina do Estado de São Paulo (protocol nº2695126), vii) ovarian cancer tissues was approved by HREC at Royal Adelaide Hospital, Adelaide, Australia (ethics #140101). Finally, adipose tissue samples from healthy donors were collected as controls after approval were obtained from the HREC at Macquarie University, Sydney, Australia (ethics #5201100385). The appropriate biosafety measures were followed while isolating and handling the human cancer cell lines and tissues.

Chemical and reagents

Ultra-pure water was from a Milli-Q system (Merck/Millipore). Recombinant *Escherichia coli* produced peptide-*N*-glycosidase F (PNGase F) from *Flavobacterium meningosepticum* and *Elizabethkingia miricola* were from Roche Diagnostics (Mannheim, Germany) and Promega (Sydney, Australia), respectively. Other chemicals, reagents and proteins were obtained from Sigma Aldrich or Thermo Fisher Scientific unless specified. Dulbecco's Modified Eagle's Medium (DMEM), MCDB 105: Medium 199 (1:1, v/v), Minimum Essential Medium (MEM alpha), Roswell Park Memorial Institute medium 1640 (RPMI), and foetal bovine serum (FBS) were from Sigma Aldrich or Thermo Fisher Scientific (Sydney, Australia).

Human cancer cell lines

Human cell lines were obtained from the American Type Culture Collection (ATCC) (Manassas, VA, USA), Cell Bank Australia (CBA) (Sydney, Australia), Cell Line Services (CLS) (Eppelheim, Germany), Garvan Institute of Medical Research (Sydney, Australia) and from multiple other sources as indicated in Supplementary Tables 1 and 2. These cell lines were collected and studied over many years by many past and present members and collaborators of our glycoanalytical laboratories as described previously [1]. The cell lines were generally stored at −140°C in 90% (v/v) FBS and 10% (v/v) dimethyl sulfoxide (DMSO) until use. All cultured cells were confirmed mycoplasma-free prior to the *N*-glycome profiling.

Tumour and non-tumour tissues from cancer patient cohorts

Tissue samples were obtained from multiple cancer patient cohorts or from various control individuals suffering from other non-cancer conditions at a range of hospitals and clinics as described in Supplementary Tables 3 and 4. Many of these tissue samples have been described in recent publications [2–11]. In brief, tumourigenic and non-tumourigenic tissues were obtained by surgery by trained clinicians after obtaining written and informed patient consent, see above and Supplementary Table 3 for ethics details.

Cell culture conditions

The human cancer cell lines were thawed and cultured in DMEM, RPMI 1640, MEM or MCDB 105 media in Corning cell culture flasks (see Supplementary Table 1 for an overview of culture conditions). These culture media differed slightly in their nutritional composition, but all contained essential components including glucose, L-glutamine, sodium pyruvate, sodium bicarbonate, phenol red as pH indicator and salts. The media were supplemented with 10% (v/v) FBS, 1% (v/v) penicillin and 100 U/ml streptomycin, and, in some cases, 25 U/l insulin, 1 μM α-thioglycerol, 1 mg/ml hydrocortisone and non-essential amino acids. FBS-free media was used to obtain

the secretome of some cultured cells (see below). All cells were grown at 37°C, 5% CO_2. The cell viabilities were regularly determined using trypan blue exclusion, counted using an automated cell counter (BioRad) and visualized using an inverted light microscope (Olympus) at 10–40x magnification to monitor the morphology of the cells. The cells were sub-cultured at 90–95% confluency typically every 72–96 h [12].

Sub-culturing of non-adherent cancer cells

Suspension cells were collected by centrifugation of the culture media at 500 × g for 10 min at 20°C. The cell pellet fractions were re-suspended in culture media and transferred to new flasks. Fresh media were added every 48 h.

Sub-culturing of adherent cancer cells

The culture media were removed, and the cells were washed with sterile-filtered phosphate buffered saline (PBS) and detached by a brief treatment with 0.25% (w/v) trypsin at 37°C. Fresh media was added (1:1, v/v) to quench the activity of trypsin and the cells were transferred to new flasks. Fresh media were added every 48 h.

For all cultured cells, the cell pellets were thoroughly washed and re-suspended in cold PBS to remove traces of FBS and then centrifuged prior to storage at −30°C until being used for analysis.

Tissue sample handling

The below describes the sample handling of the tissues obtained from patients suffering from different cancers investigated in this study.

Chronic lymphocytic leukemia (CLL)

Frozen CLL samples (~20 × 10^6 mononuclear cells) were thawed in a 37°C water bath, mixed with MACS buffer in a polypropylene round-bottom tube and centrifuged at 400 × g for 5 min at 20°C. The resulting cell pellets were re-suspended in MACS buffer, magnetically isolated using CD19-conjugated microbeads and incubated at room temperature for 15 min. The samples were again centrifuged, cell pellets were re-suspended in MACS buffer, and the B cells were isolated on MACS LS columns. The column was washed with MACS buffer to remove any unbound cells and then removed from the magnet and placed on a 15 ml CellStar tube. MACS buffer was loaded into the column and flushed out immediately by firmly pushing the plunger into the column to expel the liquid containing cells with microbeads. The process was repeating several times. The tubes were centrifuged as described above. The resulting cell pellets were lysed using probe sonication (5 s cycles, 20% amplitude) on ice and proteins were extracted by chloroform-water-methanol extraction (see below).

Basal cell carcinoma (BCC) and squamous cell carcinoma (SCC)

To allow for an accurate dermato-pathological investigation, only patients exhibiting a well-circumscribed BCC or SCC of at least 5 mm diameter were included in the study. The BCC and SCC tissues were obtained by fusiform excision with micrographic control of the margins under local anesthesia. Punch biopsies were obtained from the tumourigenic and normal regions of the excised tissues, snap-frozen in liquid nitrogen and stored at −80°C. Prior to use, the samples were washed three times with 70% (v/v) ethanol and then three times with 50 mM ammonium bicarbonate. The samples were homogenized in a lysis buffer containing 50 mM ammonium bicarbonate, 1 M urea, 10% (v/v) acetonitrile (ACN) and 0.1% (w/v) sodium dodecyl sulfate (SDS) using a IKA T10 Ultraturrax homogenizer (Staufen, Germany) and then sonicated using a tabletop Branson sonifier B-12 sonicator for 30 s. To increase the protein/peptide solubility, 4 µg trypsin was added, and the samples were incubated overnight at 37°C before insoluble material/particles were removed by centrifugation at 14,000 × g for 30 min. The supernatant fractions were collected, and the protein complement fractions were isolated by chloroform-water-methanol extraction (see below).

Hepatocellular carcinoma (HCC)

Fresh liver tumour tissues were micro-dissected and prepared for hematoxylin-eosin (H&E) staining to enable a tissue sampling containing at least 70% tumour cell content [13] before being snap-frozen in liquid nitrogen and stored at −80°C. Prior to use, the frozen tissue blocks were homogenized on ice using an IKA T10 Ultraturrax homogenizer (Staufen, Germany) in a lysis buffer containing 50 mM Tris-HCl (pH 7.4), 100 mM NaCl, and 1 mM ethylenediaminetetraacetic acid (EDTA) with 5 mM EDTA-free protease inhibitor tablets. The solutions were then sonicated on ice using a tabletop Branson sonifier B-12 sonicator for 10–30 s and centrifuged at 2,000 × g for 20 min at room temperature. The supernatant fractions were collected and the proteins isolated using microsomal preparation (see below) [14].

The formalin-fixed paraffin-embedded (FFPE) tissue samples from BCC, SCC and liver were treated with 10% (v/v) formalin for 24 h followed by washing with 70%, 95% and 100% (all v/v) ethanol (2 × 1 h each), xylene (2 × 1 h at 37°C) and then liquid paraffin (3 × 1 h at 60°C). Paraffin tissue blocks were cut into 2–10 µm thick sections using a standard sliding manual microtome (Microm, Dreieich, Germany) and transferred into 1.5 ml sample tubes, washed with xylene (2x speed for 5 min) and absolute ethanol (2x speed for 5 min) and dried. The samples were then homogenized on ice in lysis buffer containing 100 mM Tris-HCl (pH 8.0) and 100 mM dithiothreitol (DTT) and sonicated using Branson sonifier B-12 sonicator for 10–30 s. SDS (4% (w/v), final concentration) was added to the samples and incubated at 99°C for 1 h under mild agitation, cooled to ambient temperature, centrifuged at 2,000 × g for 20 min, and the resulting supernatant fractions were collected [15].

Gastric cancer (GC)

The gastric cancer patients underwent a surgical procedure with a complete resection of the tumour. From each donor, tissue samples of tumour mucosa and adjacent normal mucosa were collected and snap-frozen in liquid nitrogen (~50 mg wet weight). The tissue samples were mixed on ice in a solubilization buffer containing 7 M urea, 2 M thiourea, 40 mM Tris-HCl and a protease inhibitor cocktail mixture (1:100) and homogenized using a Precellys 24 (two cycles at power 5500, 30 s/cycle) in 2 ml reinforced homogenization tubes (Bertin Technologies, Sweden). The samples were mixed with another round of solubilization buffer with the addition of 0.5% (w/v) SDS and 10 mM DTT and left for overnight incubation on a shaker at 4°C. After overnight incubation, 25 mM iodoacetamide (IAA) was added and incubated for 40 min in the dark at room temperature. The tissue extracts were centrifuged at 19,500 × g for 20 min. The protein-containing supernatant fractions were diluted ten times with 20 mM sodium bicarbonate, applied to a preconditioned 10 kDa cut-off filter (Pall, Port Washington, USA) and washed three times in the same buffer.

Colorectal cancer (CRC)

CRC tumour samples obtained from Sydney South West Area Health Service, Australia were collected from patients undergoing surgical resection [16]. Pathological examination followed the Australian Clinico-Pathological Staging protocol for CRC, which is compatible with other staging systems e.g., the TNM (tumours/nodes/metastases) [4]. All samples were classified as T3N0M0 stage in the TNM classification. The collected CRC tissue samples were stored in Hanks' balanced salt solution at 4°C for approximately 6 h to maintain cell viability. The samples were cut into 2 mm strips and incubated in RPMI 1640 medium containing 2% (v/v) collagenase type 4 (Worthington, USA) and 0.2% (v/v) deoxyribonuclease I from bovine pancreas for 60 min at 37°C on a shaker. The semi-digested tissue was then forced gently through a fine wire mesh strainer using the plunger of a 20 ml syringe and then washed in HBSS. The resulting cell suspension was concentrated in two rounds using 200 µm and 50 µm filters to remove cell aggregates. The cell suspensions were stored at –80°C in heat-inactivated 90% (v/v) FBS and 10% (v/v) DMSO. Thawed cell suspensions were treated with 0.1% (w/v) deoxyribonuclease I and enriched for epithelial cell fractions by using immuno-magnetic beads conjugated to an antibody against the epithelial molecular marker EpCAM (Dynabead CELLection Epithelial Enrich, Invitrogen). The beads and cells were mixed and incubated for 1 h at 4°C and washed thoroughly with PBS. The proteins were extracted by acetone precipitation (see below).

CRC primary tissue samples obtained from the Department of Pathology in Yonsei University, South Korea, were diagnosed and staged for CRC by a trained pathologist at the Severance Hospital, South Korea. All CRC primary tumours were adenocarcinomas obtained from five male patients spanning differences in age and CRC pathology i.e., varying sites (sigmoid, transverse and rectum), TNM stages (I–IV), and epidermal growth factor receptor (EGFR) expression status [5, 6, 17]. The microsomal protein fractions were extracted from the tissue samples as described below.

Prostate cancer (PCa)

All biopsy cores were examined for signs of PCa by a trained pathologist. Fresh tissues were collected after radical prostatectomy from a cohort of 55 patients, which included ten patients from each disease stage (PCa grade 1–5) and five individuals presenting with benign prostatic hyperplasia (BPH). The PCa grade was defined using the Modified Gleason Grading System proposed by the International Society of Urological Pathology in 2005, a system that was revised in 2014 [18, 19]. Fresh prostate tissues (~60 mg), stored in RNALater (stabilization and storage solution), were washed in 80% (v/v) cold ACN and re-suspended in an extraction buffer containing 6 M urea, 10 mM DTT, 1 mM sodium fluoride, 1 mM sodium orthovanadate and a protease inhibitor cocktail (1:10). Prostate tissues were rapidly lysed (2 min, 30 Hz with a 5 mm stainless steel bead) using a TissueLyser (Qiagen, Chadstone, VIC, Australia). Protein concentrations were determined by the Qubit fluorimetric detection method. The protein samples were directly used for *N*-glycomics.

Protein extracts from cellular fractions

As briefly described below, protein extracts from different cellular fractions were obtained from the investigated cell lines and tissue samples.

Secretome (S)

Cells grown to high (>95%) confluency were washed at least four times with ice-cold PBS to remove FBS and incubated in FBS-free media at 37°C in 5% CO_2 for 28–48 h. The media containing the secreted proteins were collected and centrifuged at 2,000 × g to pellet any cells or cellular debris. The proteins in the supernatant fractions (hereafter the "secretome") were transferred to new vials and concentrated using a 10 kDa molecular weight cut-off Amicon Ultra Centrifugal filter device (Millipore, USA) and stored in 4°C until use.

Whole cell lysate (WCL)

Cell pellets (see above) were gently thawed on ice, re-suspended in radioimmunoprecipitation assay lysis buffer, vigorously vortexed and centrifuged at 14,000 × g, 4°C for 10–20 min. The resulting supernatant fractions (the "whole cell lysates") containing the cellular proteins were collected and stored at 4°C until use.

Microsomal fraction (MF)

This preparation follows a previously published protocol [20]. In short, cell pellets were re-suspended

in 25 mM Tris-HCl (pH 7.4), 150 mM NaCl, and 1 mM EDTA with 5 mM EDTA-free protease inhibitor tablets. The cell suspension was ultra-sonicated using Sonifier 450 on ice over three cycles of 10 s bursts and then centrifuged at 2,000 × g, 4°C for 20 min. The resulting supernatant was ultra-centrifuged at 120,000 × g, 4°C for 80 min. The resulting pellet containing the total membrane complement was re-suspended in 25 mM Tris-HCl (pH 7.4), 150 mM NaCl and 1% (v/v) Triton X-114 and phase partitioned on ice for 10 min, and then at 37°C for 20 min. A subsequent gentle centrifugation step at 1,000 × g at 25°C for 10 min created two distinct phases. The dense detergent phase containing the membrane protein complement (the "microsome") was stored in 4°C until use.

Protein isolation

Acetone precipitation

The proteins of all three cellular fractions (WCL, MF, and S, see above) from most cells and tissues investigated in this study were precipitated by adding ice-cold acetone in a ratio of 1:4–1:9 (v/v), vortexed thoroughly and incubated overnight at −20°C in an upright position. The samples were then centrifuged at 14,000 × g for 12–15 min and the protein pellets were stored in −30°C until further analysis.

Chloroform-water-methanol extraction

The proteins in the supernatant fractions after cell lysis of CLL tissues (B cells) and non-melanoma BCC tissues were precipitated according to the method used by Wessel and Flugge [21]. Briefly, the supernatant was mixed vigorously with methanol, chloroform and water in a 4:1:3 volume ratio. The samples were centrifuged at 14,000 × g for 5 min and the upper aqueous phase was carefully removed. Additional methanol was added, mixed and centrifuged as above. The supernatant was removed, and dried protein pellet was stored at −20°C until analysis.

The protein pellets were re-suspended in 6-8 M urea and the protein concentrations were determined using the Bradford assay or the bicinchoninic acid assay.

Protein denaturation

Proteins were reduced using 10 mM DTT, 45 min, 56°C, and carbamidomethylated using 25 mM IAA, 30 min in the dark at 20°C before the alkylation reactions were quenched using 30 mM DTT (final concentrations stated).

Abbreviations

ACN: acetonitrile; ALL: acute lymphocytic leukemia; AML: acute monocytic leukemia; APL: acute promyelocytic leukemia; ATCC: American Type Culture Collection; BC: breast cancer; BCC: basal cell carcinoma; BlaCa: bladder cancer; BPH: benign prostatic hyperplasia; BSA: bovine serum albumin; CBA: Cell Bank Australia; CC: cholangiocarcinoma; CLL: chronic lymphocyte leukemia; CLS: Cell Line Services; ConA: concanavalin A; CRC: colorectal cancer; DMEM: Dulbecco's Modified Eagle's Medium; DMSO: dimethyl sulfoxide; DTT: dithiothreitol; EDTA: ethylenediaminetetraacetic acid; ER: endoplasmic reticulum; FBS: fetal bovine serum; FF: fresh frozen; FFPE: formalin-fixed paraffin-embedded; Fuc: fucose; Gal: galactose; GC: gastric cancer; GEO: Gene Expression Omnibus; Glc: glucose; GlcNAc: *N*-acetylglucosamine; H&E: hematoxylin-eosin; HCC: hepatocellular carcinoma; HREC: human research ethics committee; IAA: iodoacetamide; KC: kidney cancer; LC-MS/MS: liquid chromatography tandem mass spectrometry; MALDI-MSI: matrix-assisted laser desorption/ionization mass spectrometry imaging; Man: mannose; Me-a-Man: methyl-a-D-mannopyranoside; MEM: Minimum Essential Medium; MF: microsomal fraction; NeuAc: *N*-acetylneuraminic acid; OC: oral cancer; OvC: ovarian cancer; P: paired tissue; PanCa: pancreas cancer; PBS: phosphate buffered saline; PCa: prostate cancer; PGC: porous graphitized carbon; RPMI: Roswell Park Memorial Institute Medium; S: secretome; SCC: squamous cell carcinoma; SD: standard deviation; SDS: sodium dodecyl sulfate; TC: thyroid cancer; TCGA: The Cancer Genome Atlas; TMA: tissue microarray; WCL: whole cell lysate.

SUPPLEMENTARY REFERENCES

1. Chatterjee S, Lee LY, Kawahara R, Abrahams JL, Adamczyk B, Anugraham M, Ashwood C, Sumer-Bayraktar Z, Briggs MT, Chik JHL, Everest-Dass A, Förster S, Hinneburg H, et al. Protein Paucimannosylation Is an Enriched N-Glycosylation Signature of Human Cancers. Proteomics. 2019; 19:e1900010. https://doi.org/10.1002/pmic.201900010. [PubMed]
2. Möginger U, Grunewald S, Hennig R, Kuo CW, Schirmeister F, Voth H, Rapp E, Khoo KH, Seeberger PH, Simon JC, Kolarich D. Alterations of the Human Skin *N*- and *O*-Glycome in Basal Cell Carcinoma and Squamous Cell Carcinoma. Front Oncol. 2018; 8:70. https://doi.org/10.3389/fonc.2018.00070. [PubMed]
3. Lee LY, Thaysen-Andersen M, Baker MS, Packer NH, Hancock WS, Fanayan S. Comprehensive N-glycome profiling of cultured human epithelial breast cells identifies unique secretome N-glycosylation signatures enabling tumorigenic subtype classification. J Proteome Res. 2014; 13:4783–95. https://doi.org/10.1021/pr500331m. [PubMed]
4. Chik JH, Zhou J, Moh ES, Christopherson R, Clarke SJ, Molloy MP, Packer NH. Comprehensive glycomics comparison between colon cancer cell cultures and tumours: implications for biomarker studies. J Proteomics. 2014; 108:146–62. https://doi.org/10.1016/j.jprot.2014.05.002. [PubMed]
5. Sethi MK, Kim H, Park CK, Baker MS, Paik YK, Packer NH, Hancock WS, Fanayan S, Thaysen-Andersen M. In-

depth N-glycome profiling of paired colorectal cancer and non-tumorigenic tissues reveals cancer-, stage- and EGFR-specific protein N-glycosylation. Glycobiology. 2015; 25:1064–78. https://doi.org/10.1093/glycob/cwv042. [PubMed]

6. Sethi MK, Thaysen-Andersen M, Kim H, Park CK, Baker MS, Packer NH, Paik YK, Hancock WS, Fanayan S. Quantitative proteomic analysis of paired colorectal cancer and non-tumorigenic tissues reveals signature proteins and perturbed pathways involved in CRC progression and metastasis. J Proteomics. 2015; 126:54–67. https://doi.org/10.1016/j.jprot.2015.05.037. [PubMed]
7. Nakano M, Saldanha R, Göbel A, Kavallaris M, Packer NH. Identification of glycan structure alterations on cell membrane proteins in desoxyepothilone B resistant leukemia cells. Mol Cell Proteomics. 2011; 10:M111.009001. https://doi.org/10.1074/mcp.M111.009001. [PubMed]
8. Hinneburg H, Korać P, Schirmeister F, Gasparov S, Seeberger PH, Zoldoš V, Kolarich D. Unlocking Cancer Glycomes from Histopathological Formalin-fixed and Paraffin-embedded (FFPE) Tissue Microdissections. Mol Cell Proteomics. 2017; 16:524–36. https://doi.org/10.1074/mcp.M116.062414. [PubMed]
9. Abrahams JL, Packer NH, Campbell MP. Relative quantitation of multi-antennary N-glycan classes: combining PGC-LC-ESI-MS with exoglycosidase digestion. Analyst. 2015; 140:5444–49. https://doi.org/10.1039/c5an00691k. [PubMed]
10. Anugraham M, Jacob F, Nixdorf S, Everest-Dass AV, Heinzelmann-Schwarz V, Packer NH. Specific glycosylation of membrane proteins in epithelial ovarian cancer cell lines: glycan structures reflect gene expression and DNA methylation status. Mol Cell Proteomics. 2014; 13:2213–32. https://doi.org/10.1074/mcp.M113.037085. [PubMed]
11. Kawahara R, Ortega F, Rosa-Fernandes L, Guimarães V, Quina D, Nahas W, Schwämmle V, Srougi M, Leite KRM, Thaysen-Andersen M, Larsen MR, Palmisano G. Distinct urinary glycoprotein signatures in prostate cancer patients. Oncotarget. 2018; 9:33077–97. https://doi.org/10.18632/oncotarget.26005. [PubMed]
12. Phelan K, May KM. Basic techniques in mammalian cell tissue culture. Curr Protoc Cell Biol. 2015; 66:1.1.1–1.22. https://doi.org/10.1002/0471143030.cb0101s66. [PubMed]
13. Fischer AH, Jacobson KA, Rose J, Zeller R. Hematoxylin and eosin staining of tissue and cell sections. CSH Protoc. 2008; 2008:pdb.prot4986. https://doi.org/10.1101/pdb.prot4986. [PubMed]
14. Lee A, Kolarich D, Haynes PA, Jensen PH, Baker MS, Packer NH. Rat liver membrane glycoproteome: enrichment by phase partitioning and glycoprotein capture. J Proteome Res. 2009; 8:770–81. https://doi.org/10.1021/pr800910w. [PubMed]
15. Ostasiewicz P, Zielinska DF, Mann M, Wiśniewski JR. Proteome, phosphoproteome, and N-glycoproteome are quantitatively preserved in formalin- fixed paraffin-embedded tissue and analyzable by high-resolution mass spectrometry. J Proteome Res. 2010; 9:3688–700. https://doi.org/10.1021/pr100234w. [PubMed]
16. Zhou J, Belov L, Huang PY, Shin JS, Solomon MJ, Chapuis PH, Bokey L, Chan C, Clarke C, Clarke SJ, Christopherson RI. Surface antigen profiling of colorectal cancer using antibody microarrays with fluorescence multiplexing. J Immunol Methods. 2010; 355:40–51. https://doi.org/10.1016/j.jim.2010.01.015. [PubMed]
17. Sethi MK, Thaysen-Andersen M, Smith JT, Baker MS, Packer NH, Hancock WS, Fanayan S. Comparative N-glycan profiling of colorectal cancer cell lines reveals unique bisecting GlcNAc and α-2,3-linked sialic acid determinants are associated with membrane proteins of the more metastatic/aggressive cell lines. J Proteome Res. 2014; 13:277–88. https://doi.org/10.1021/pr400861m. [PubMed]
18. Kryvenko ON, Epstein JI. Prostate Cancer Grading: A Decade After the 2005 Modified Gleason Grading System. Arch Pathol Lab Med. 2016; 140:1140–52. https://doi.org/10.5858/arpa.2015-0487-SA. [PubMed]
19. Borghesi M. Ruolo della risonanza magnetica multiparametrica nella selezione dei pazienti con carcinoma prostatico a rischio molto basso candidabili a sorveglianza attiva: studio prospettico. Alma. 2018. https://doi.org/10.6092/unibo/amsdottorato/8375.
20. Hua S, Saunders M, Dimapasoc LM, Jeong SH, Kim BJ, Kim S, So M, Lee KS, Kim JH, Lam KS, Lebrilla CB, An HJ. Differentiation of cancer cell origin and molecular subtype by plasma membrane N-glycan profiling. J Proteome Res. 2014; 13:961–68. https://doi.org/10.1021/pr400987f. [PubMed]
21. Wessel D, Flügge UI. A method for the quantitative recovery of protein in dilute solution in the presence of detergents and lipids. Anal Biochem. 1984; 138:141–43. https://doi.org/10.1016/0003-2697(84)90782-6. [PubMed]
22. Moremen KW, Tiemeyer M, Nairn AV. Vertebrate protein glycosylation: diversity, synthesis and function. Nat Rev Mol Cell Biol. 2012; 13:448–62. https://doi.org/10.1038/nrm3383. [PubMed]
23. Hare NJ, Lee LY, Loke I, Britton WJ, Saunders BM, Thaysen-Andersen M. Mycobacterium tuberculosis Infection Manipulates the Glycosylation Machinery and the N-Glycoproteome of Human Macrophages and Their Microparticles. J Proteome Res. 2017; 16:247–63. https://doi.org/10.1021/acs.jproteome.6b00685. [PubMed]
24. Kawahara R, Recuero S, Srougi M, Leite KRM, Thaysen-Andersen M, Palmisano G. The Complexity and Dynamics of the Tissue Glycoproteome Associated With Prostate Cancer Progression. Mol Cell Proteomics. 2021; 20:100026. https://doi.org/10.1074/mcp.RA120.002320. [PubMed]

Annotation and fragmentation key

- Mannose (Man) (162.0528 Da)
- Glucose (Glc) (162.0528 Da)
- *N*-Acetylglucosamine (GlcNAc) (203.0794 Da)
- Cross-ring fragment (unspecified)
- Indicates both Y and B ions (includes oxygen of glycosidic linkage)
- Indicates both Z and C ions (excludes oxygen of glycosidic linkage)
- Reduced reducing end

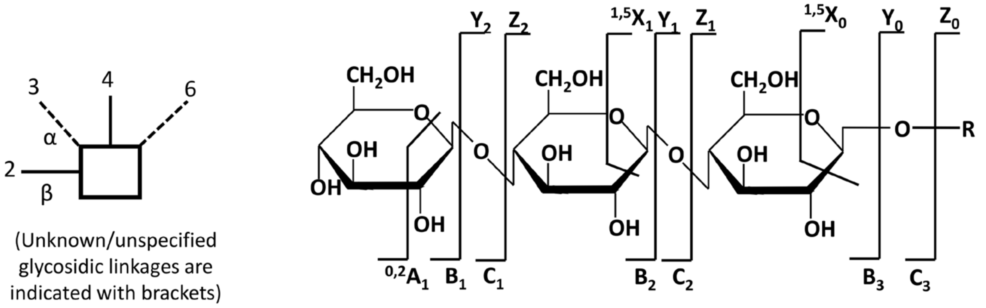

M5a: Man$_5$GlcNAc$_2$ (isomer a)
Observed *m/z* 1235.56 (1-), RT: ~23.4 min

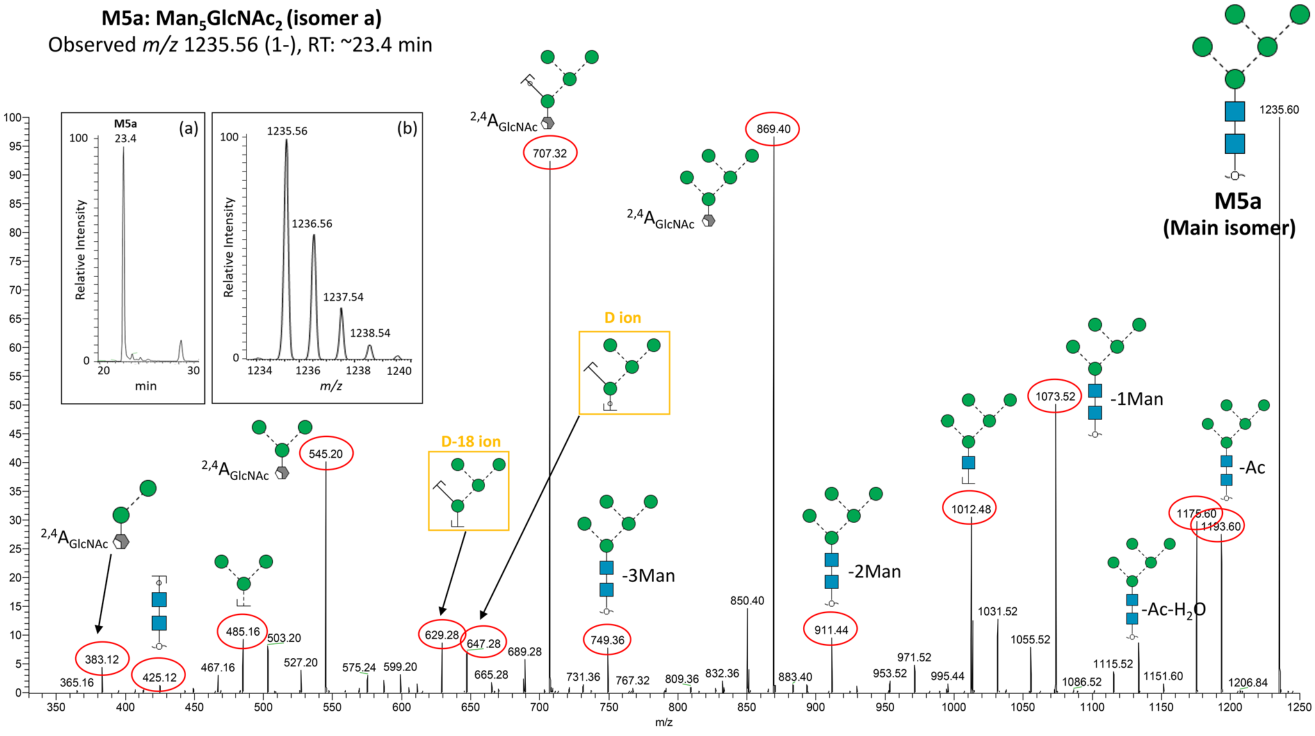

M5b: $Man_5GlcNAc_2$ (isomer b)

Observed *m/z* 1235.56 (1-), RT: ~28.9 min

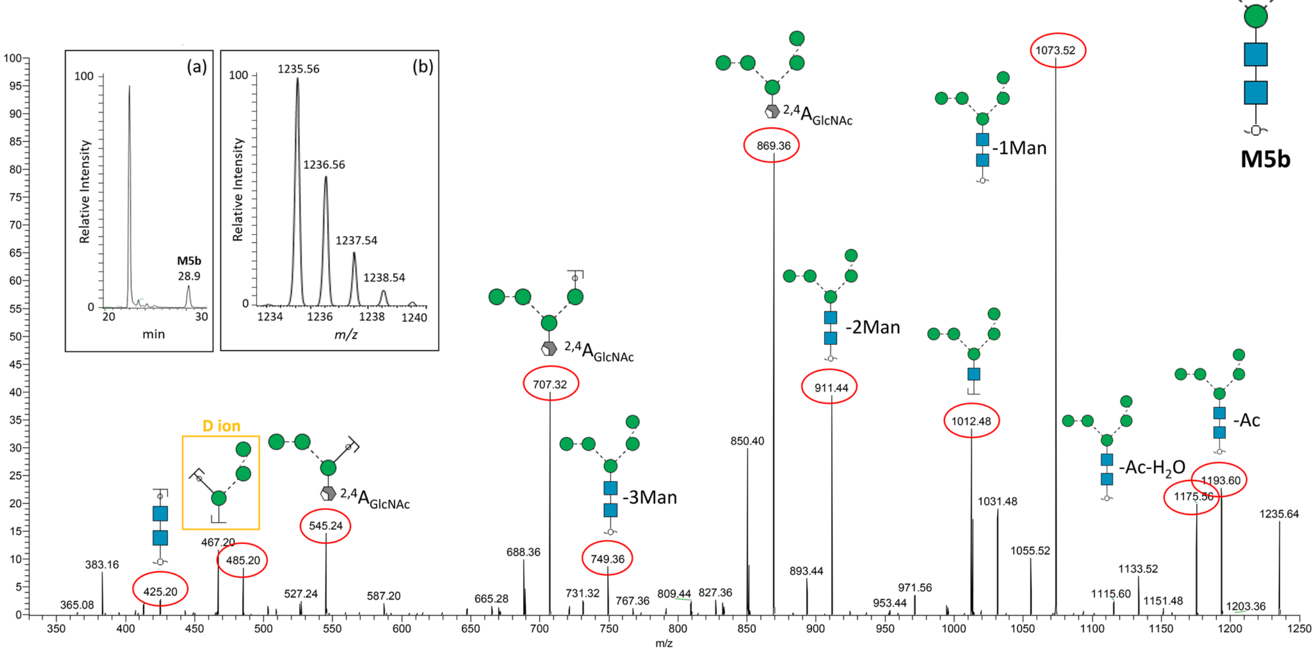

M6a: $Man_6GlcNAc_2$ (isomer a)

Observed *m/z* 698.28 (2-), RT: ~18.3 min

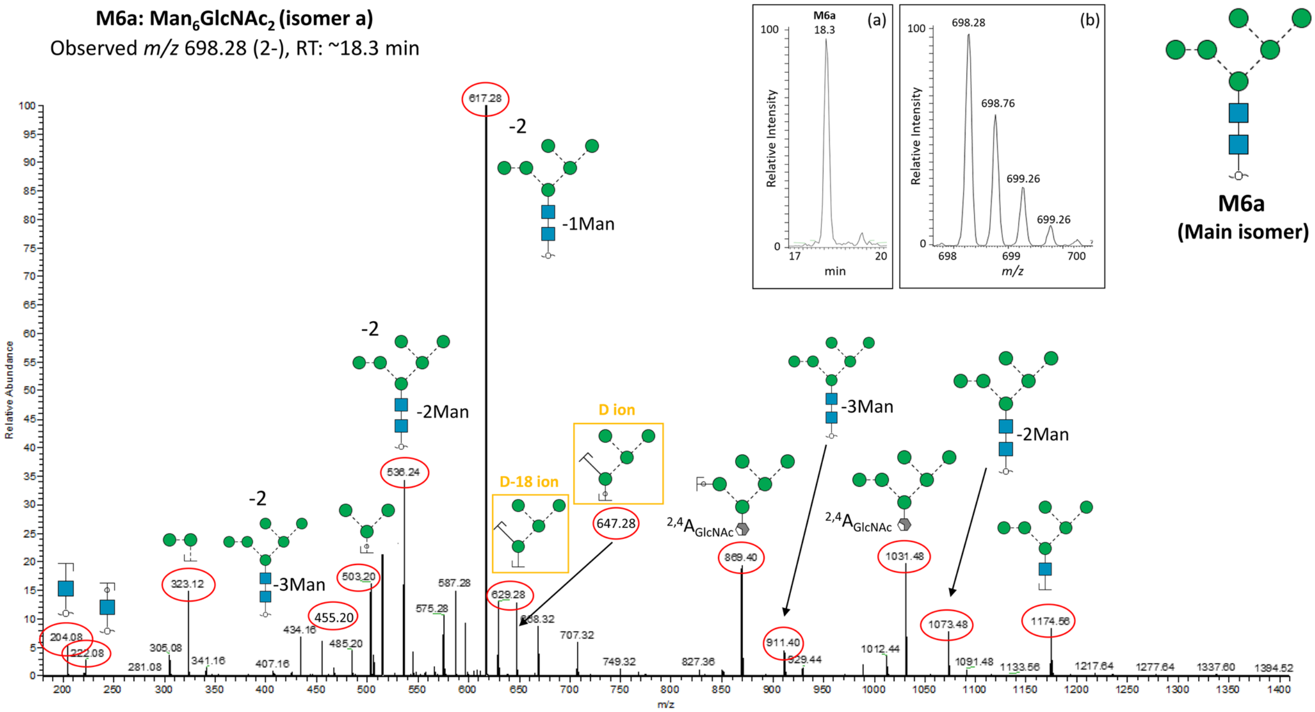

M6b: Man$_6$GlcNAc$_2$ (isomer b)

Observed *m/z* 698.28 (2-), RT: ~19.5 min

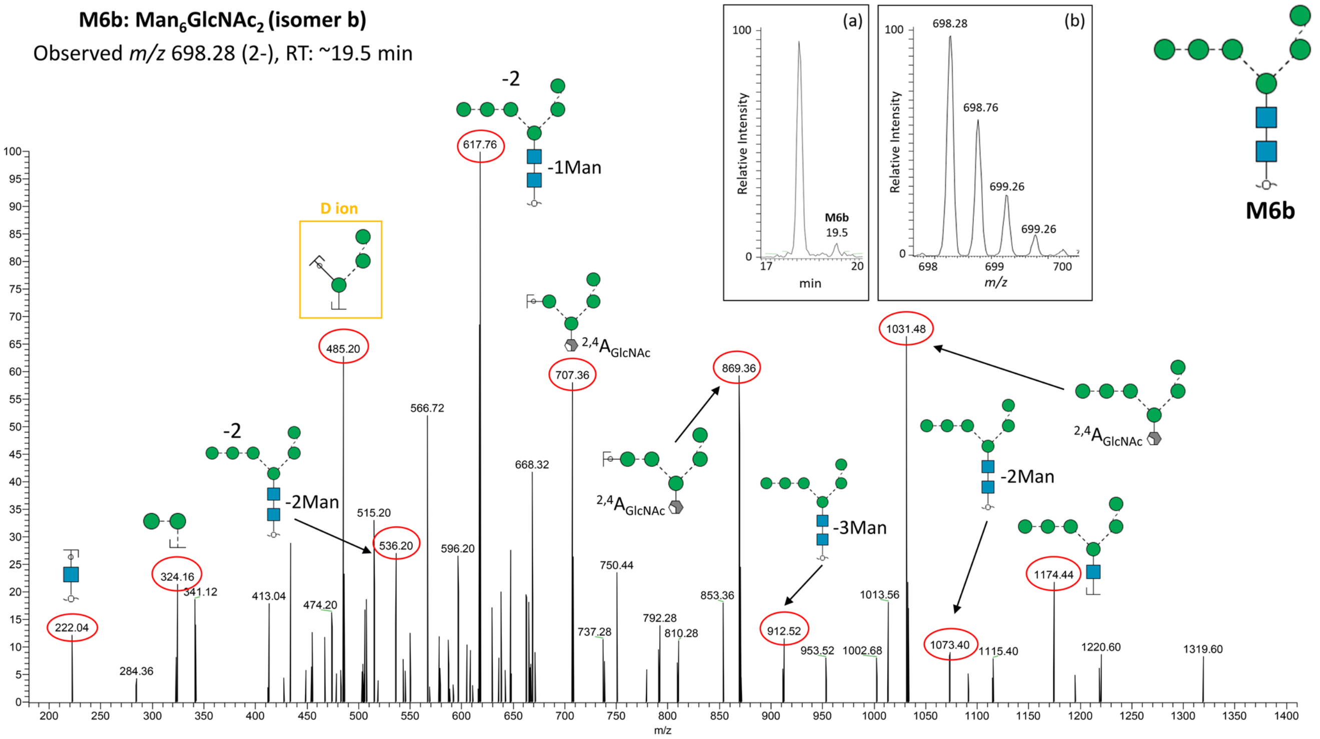

M7a: Man$_7$GlcNAc$_2$ (isomer a)

Observed *m/z* 779.30 (2-), RT: ~17.8 min

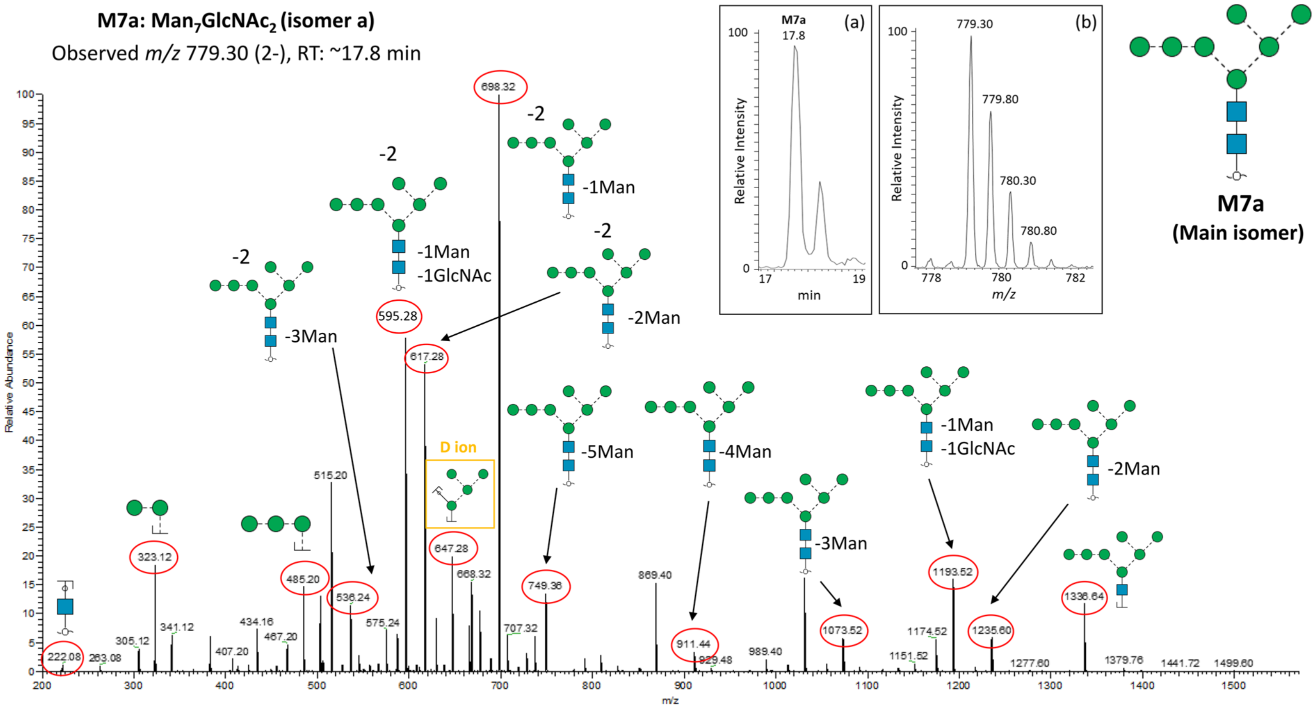

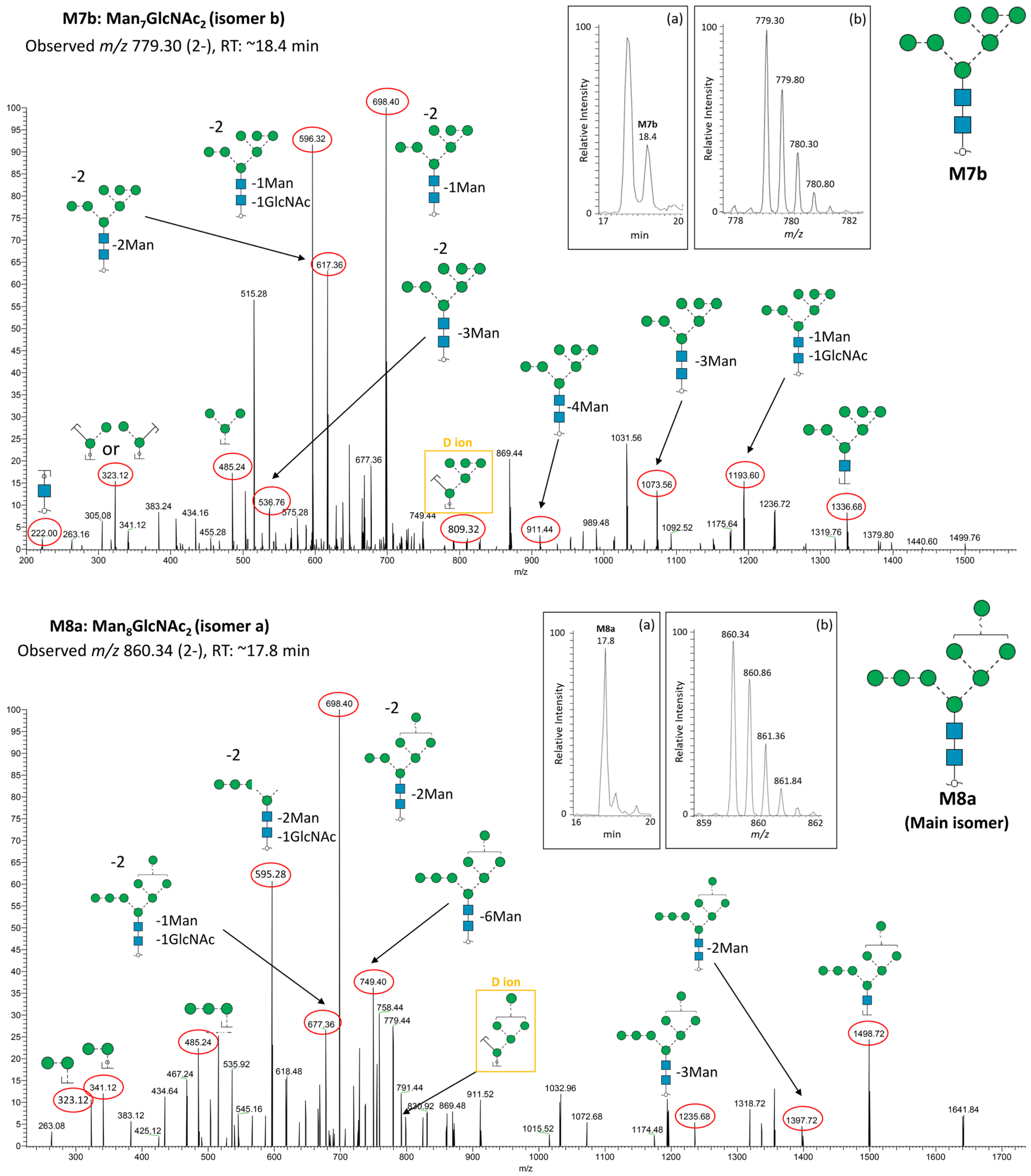
M7b: Man$_7$GlcNAc$_2$ (isomer b)
Observed *m/z* 779.30 (2-), RT: ~18.4 min
(a)
Relative Intensity
M7b
18.4
min
(b)
779.30
779.80
780.30
780.80
m/z
M7b
-2
-2Man
-1Man
-1GlcNAc
-3Man
or
D ion
-4Man
-1Man
-1GlcNAc
222.00
263.16
305.08
323.12
341.12
383.24
434.16
455.28
485.24
515.28
536.76
575.28
596.32
617.36
677.36
698.40
749.44
809.32
869.44
911.44
989.48
1031.56
1073.56
1092.52
1175.64
1193.60
1236.72
1319.76
1336.68
1379.80
1440.60
1499.76
m/z
M8a: Man$_8$GlcNAc$_2$ (isomer a)
Observed *m/z* 860.34 (2-), RT: ~17.8 min
(a)
M8a
17.8
Relative Intensity
min
(b)
860.34
860.86
861.36
861.84
m/z
M8a
(Main isomer)
-2
-2Man
-1GlcNAc
-1Man
-1GlcNAc
-6Man
-2Man
-3Man
D ion
263.08
323.12
341.12
383.12
425.12
434.64
467.24
485.24
535.92
545.16
595.28
618.48
677.36
698.40
749.40
758.44
779.44
791.44
830.92
869.48
911.52
1015.52
1032.96
1072.68
1174.48
1235.68
1318.72
1397.72
1498.72
1641.84
m/z

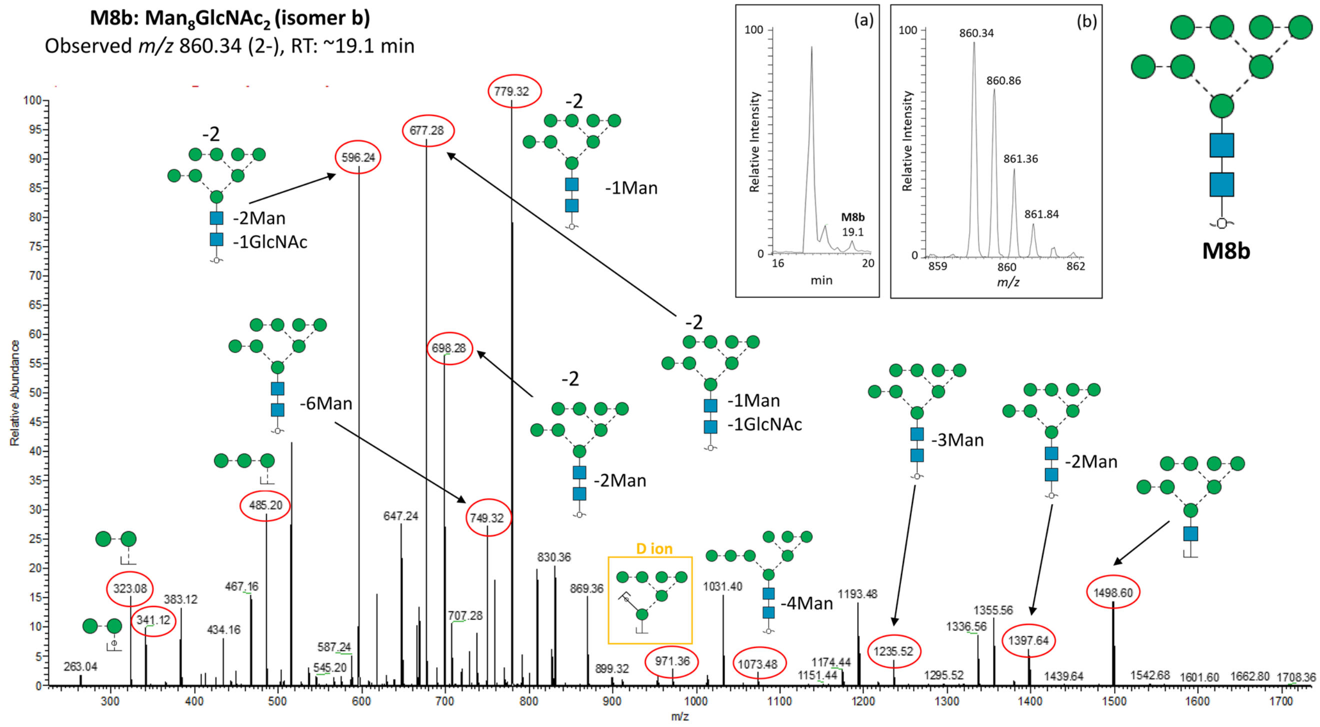

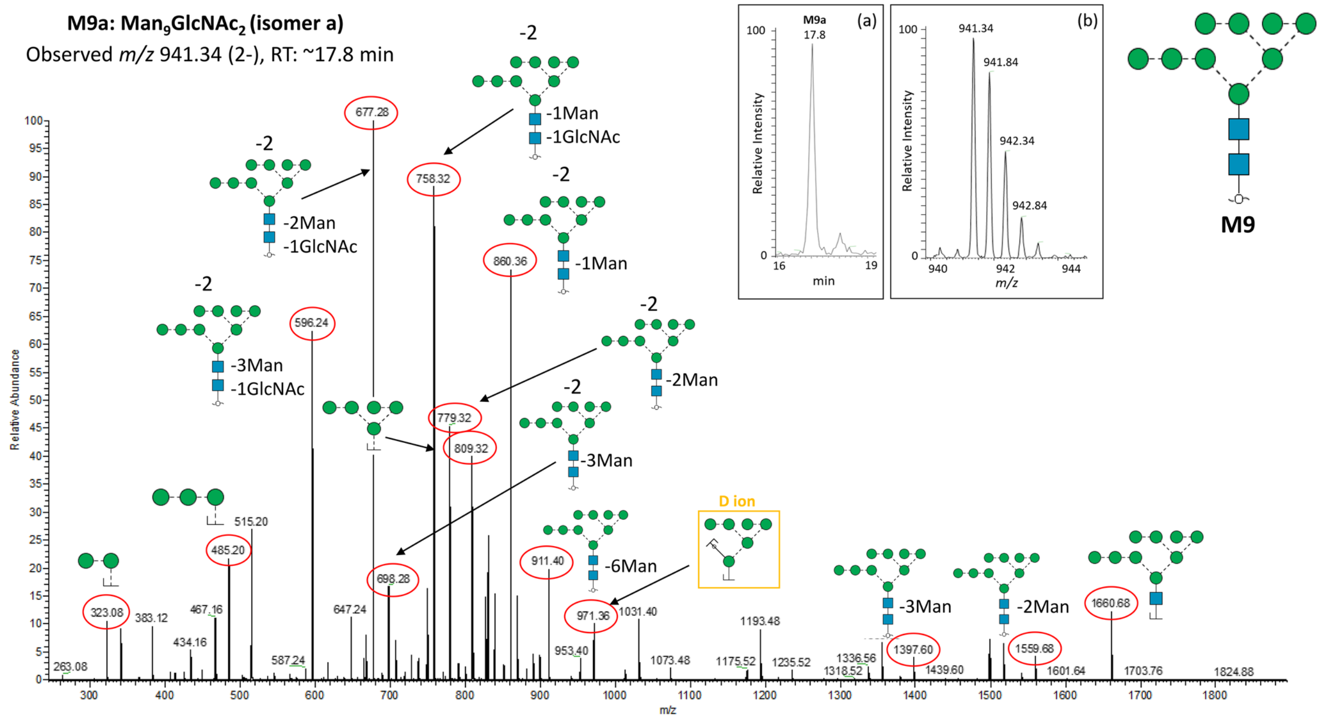

Supplementary Data 1: CID-MS/MS spectral evidence and PGC-LC retention time information of all oligomannosidic *N*-glycans species (M5-M9) reported in this study. The PGC-LC-ESI-CID-MS/MS (−) spectra were generated from reduced *N*-glycans (alditols) liberated from protein extracts of human cancer cell lines and tissues. Representative MS/MS spectra have been manually annotated and MS1 spectra focusing on the precursorion envelope (**a**) and the PGC-LC retention time and relative isomer abundance as shown by precursor ion EICs (**b**) have been provided, see inserts. Note the abundance of the isomeric structures observed for some oligomannosidic *N*-glycan species (M5-M9 as documented with the enclosed spectra) were combined for the relative quantitation.

Supplementary Data 2: Quantitation of *N*-glycans in human cultured cancer cell lines (data plotted in Figure 2). See Supplementary Data 2

Supplementary Data 3: Quantitation of *N*-glycans in human cancer tissues (data plotted in Figure 3 and Supplementary Figure 1). See Supplementary Data 3

Supplementary Data 4A: Transcript levels of MAN1A1, MAN1A2, MAN1B1, and MAN1C1 as extracted from the gene expression omnibus (GEO) database (data plotted in Figure 4). See Supplementary Data 4A

Supplementary Data 4B: Reinterrogation of data from publicly available transcriptomics database (CellMiner™) database (data plotted in Supplementary Figure 3A). See Supplementary Data 4B

Supplementary Data 4C: Reinterrogation of data from publicly available genomics (TCGA) database (data plotted in Supplementary Figure 3B). See Supplementary Data 4C

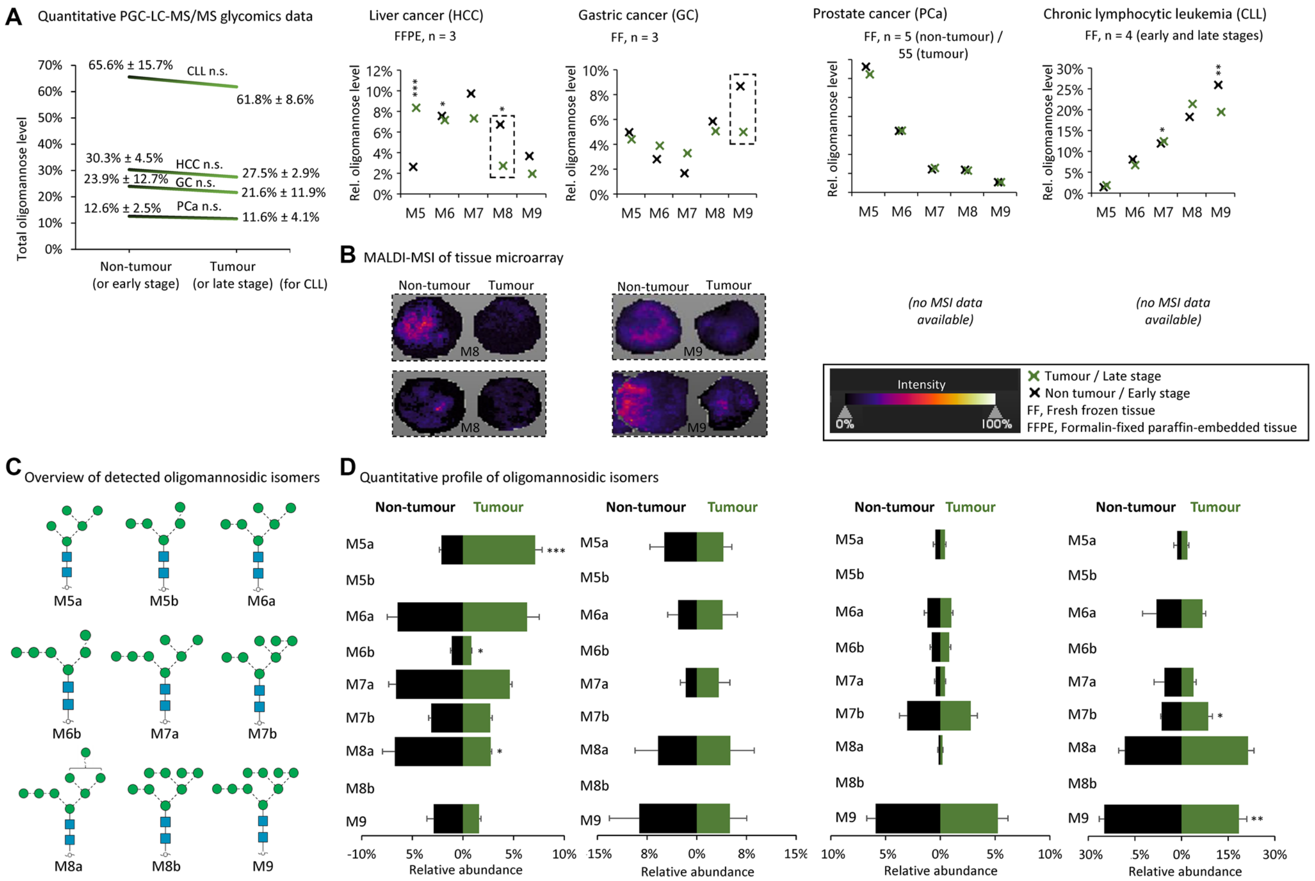

Supplementary Figure 1: Tissue glycomics and MALDI-MSI data suggest that a subset of cancers do not display oligomannose elevation. (**A**) A subset of cancers did not exhibit oligomannose elevation as assessed via quantitative glycomics of paired tumour and non-tumour tissues from patients with liver cancer (HCC) and gastric cancer (GC), and of unpaired tumour and non-tumour tissues from patients with prostate cancer (PCa) and patients with early- or late-stage chronic lymphocytic leukemia (CLL). See Supplementary Tables 3 and 4 for details of the investigated tissues. See Supplementary Data 3 and Supplementary Tables 8 and 9 for all raw and tabulated glycome data, respectively. Statistics was performed using paired and unpaired two-tailed *t* tests (*n*, patient samples, as indicated). $^{*}p < 0.05$, $^{**}p < 0.001$, $^{***}p < 0.0001$, n.s., not significant ($p \geq 0.05$). (**B**) The MALDI-MSI data were from a TMA containing unpaired tumour and non-tumour tissues. Representative examples of oligomannosidic *N*-glycans displaying prominent regulation in the tumour tissues have been provided (boxed, broken line). No MSI data could be obtained for PCa and CLL since these tissues were not part of the sourced TMA. See key for MALDI-MSI intensity range and Supplementary Table 5 for details of the TMA used for the MALDI-MSI experiment. (**C**) Overview of the oligomannosidic isomers detected from the PGC-LC-MS/MS glycomics experiments. (**D**) Quantitative profile of the detected oligomannosidic isomers from tumour and non-tumour tissues of the cancer types explored in (A). Statistics was performed using paired and unpaired two-tailed *t* tests (*n*, patient samples, as indicated). $^{*}p < 0.05$, $^{**}p < 0.001$, $^{***}p < 0.0001$, n.s. not significant ($p \geq 0.05$).

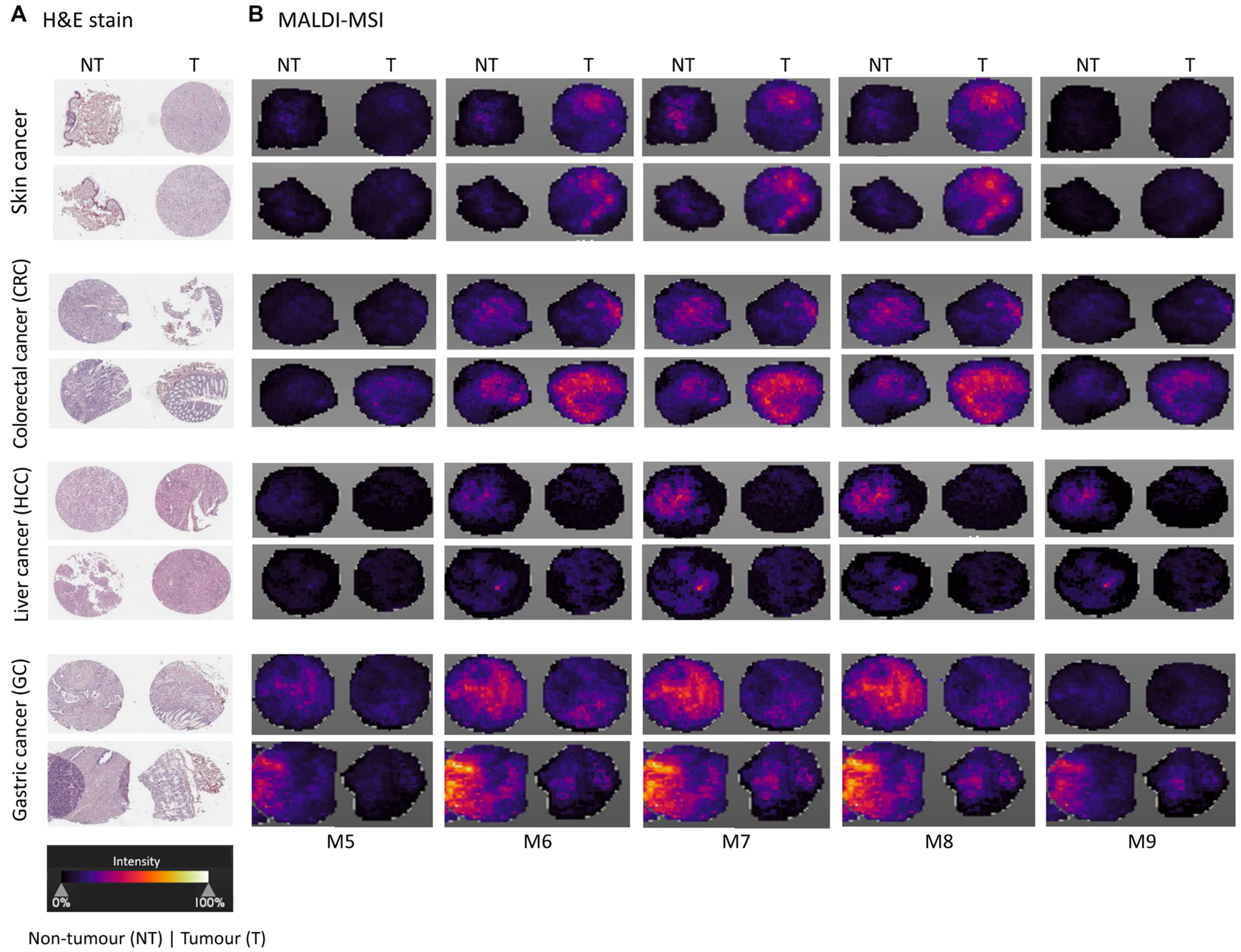

Supplementary Figure 2: TMA-based MALDI-MSI data of four cancer types with matching glycomics data. (**A**) H&E staining and (**B**) MALDI-MSI data of the entire M5-M9 oligomannosidic *N*-glycan series from skin, colorectal (CRC), liver (HCC) and gastric (GC) cancers investigated in this study. While the TMA also contained tissues from other cancer types, only data from these four types of cancer were shown since they have matching glycomics data, see Figure 3 and Supplementary Figure 1. See key for MALDI-MSI intensity range and Supplementary Table 5 for details of the TMA used for the MALDI-MSI experiment.

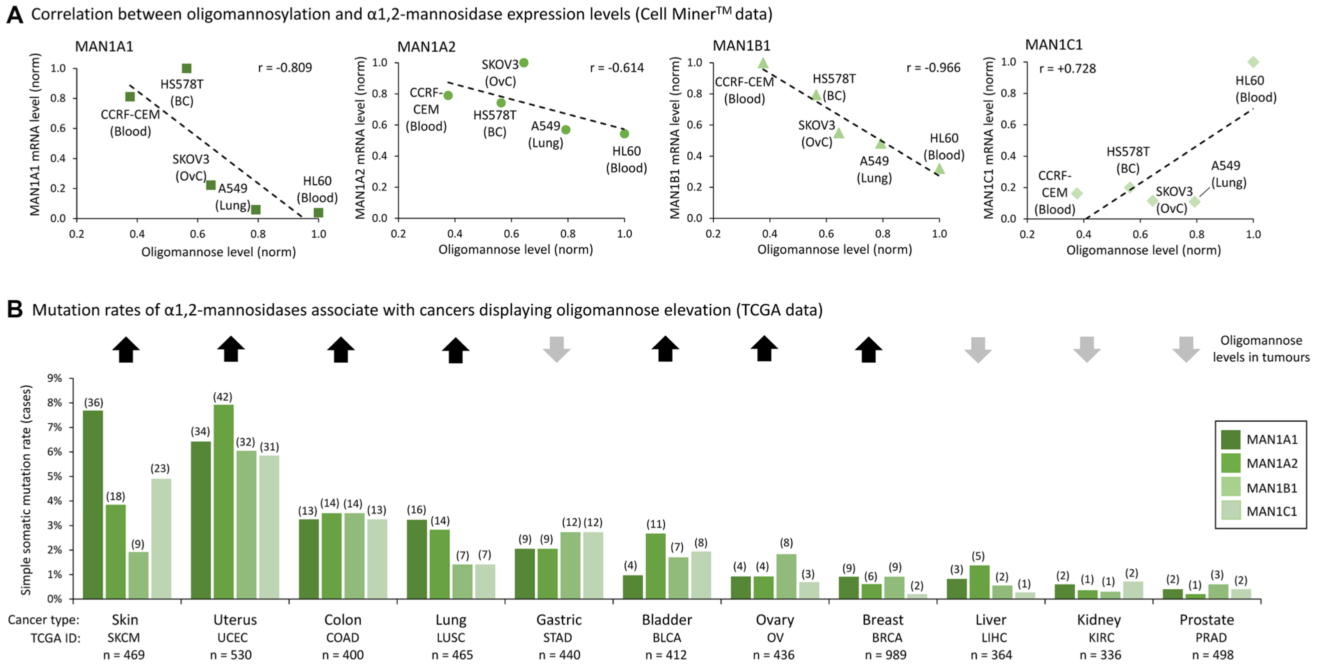

Supplementary Figure 3: Altered expression of α1,2-mannosidase and oligomannose in cancer. (**A**) Correlation analysis between the expression (mRNA) of the four α1,2-mannosidases (MAN1A1, MAN1A2, MAN1B1, and MAN1C1 [22], see Figure 1), as extracted from the Cell Miner™ database, and the total oligomannose levels in five cancer cell lines covered by both our glycomics data (Figure 2) and the Cell Miner™ database (Supplementary Data 4B). MAN1A1, MAN1A2, and MAN1B1 showed a negative correlation with the total oligomannose levels. (**B**) Frequency of simple somatic mutations of MAN1A1, MAN1A2, MAN1B1 and MAN1C1 determined based on data retrieved from The Cancer Genome Atlas (TCGA) across 11 cancer types investigated in this study (Supplementary Data 4C). The up- (black arrows) and down-regulation/unchanged levels (grey arrows) of oligomannose in tumours based on literature (Table 1) and/or our own glycomics data (Figure 3 and Supplementary Figure 1) are indicated for each cancer type to aid the data interpretation. The number of affected cases is indicated for each of the four α1,2-mannosidases in each cohort across the investigated cancer types.

Supplementary Table 1: Overview of the cultured cell lines, their culture conditions and protein fraction(s) analyzed in this study

Cancer type	Primary contributor#	Cell lines (origin/identifier)	Culture media and properties	Protein fraction	Ref
**1. Brain cancer**					
A. Glioblastoma	CA	i. U87MG<br>(ATCC HTB-14)	DMEM, adherent	WCL	In prep
	IL	i. U87MG[a]<br>(ATCC HTB-14)<br>ii. A172[a]<br>(ATCC CRL-1620)<br>iii. U118MG<br>(CLS 300362)<br>iv. U138MG[a]<br>(CLS 300363)	DMEM, adherent	MF<br>MF, S, WCL<br>MF, WCL<br>MF, WCL	In prep
B. Neuroblastoma	ZSB	i. SK-N-B(2)[b]<br>(ATCC CRL-2271)	DMEM, adherent	MF	In prep
**2. Blood cancer**					
A. APL	IL	i. HL-60 (ATCC CCL-240)	RPMI 1640, suspension	Differentiated/undifferentiated WCL	-
B. AML	LYL	i. THP1<br>(ATCC TIB-202)	RPMI 1640, suspension	Uninfected /infected MF, WCL	[23]
C. ALL	MN	i. CCRF-CEM<br>(T-cell)<br>(ATCC CCL-119)	RPMI 1640, suspension	MF	[7]
**3. Melanoma skin cancer**	JLA	i. MM253<br>(CBA-1347)	RPMI 1640, adherent	MF	[9]
**4. BC**	LYL	i. HMEC[*]<br>(ATCC CRL-3243)<br>ii. HS578T<br>(ATCC HTB-126)<br>iii. MCF7<br>(ATCC HTB-22)<br>iv. MDA-MB157<br>(ATCC HTB-24)<br>v. MDA-MB231<br>(ATCC HTB-26)<br>vi. MDA-MB468<br>(ATCC HTB-132)<br>vii. SKBR3<br>(ATCC HTB-30)	RPMI 1640, adherent	MF, S	[3]
**5. Lung cancer**	EM	i. A549<br>(ATCC CCL-185)	RPMI 1640, adherent	MF, WCL	-
**6. HCC**	SC	i. HepG2<br>(ATCC HB-8065)	DMEM, adherent	MF, WCL	-
**7. CRC**	JHLC	i. SW480<br>(ATCC CCL-228)<br>ii. SW620<br>(ATCC CCL-227)<br>iii. SW837<br>(ATCC CCL-235)<br>iv. SW1116<br>(ATCC CCL-233)	DMEM, adherent	MF	[4]
	MKS	v. LIM1215[c]<br>vi. LIM1899[c]<br>vii. LIM2405[c]	RPMI 1640, adherent	MF	[5]
**8. OvC**	MA	i. A2780<br>(ATCC CRL-2772)<br>ii. IGROV1<br>(ATCC TCP-1021)<br>iii. OVCAR3<br>(ATCC HTB-161)<br>iv. SKOV3<br>(ATCC HTB-77)<br>v. HOSE 6.3[*d]<br>vi. HOSE 17.1[*d]	i-iv) RPMI 1640, adherent<br>v-vi) MCDB 105: medium 199, adherent	MF	[10]
**9. BlaCa**	EM	i. J82<br>(ATCC HTB-1)<br>ii. RT112<br>(ATCC TCP-1020)<br>iii. UROtsa[*e]	i) MEM alpha media<br>ii-iii) RPMI 1640 adherent	MF, WCL	-

[a]Gift from Prof Torsten Pietsch, Institute of Neuropathology, University of Bonn Medical Center, Bonn, Germany. [b]Gift from Prof Maria Kavallaris, Children's Cancer Institute, Lowy Cancer Research Centre, University of New South Wales, Sydney, NSW, Australia. [c]Ludwig Institute for Cancer Research, Melbourne, VIC, Australia. [d]Garvan Institute of Medical Research, Sydney, NSW, Australia. [e]Gift from Prof Philip Erben, Department of Urology, Medical Faculty Mannheim, Heidelberg University, Mannheim, Germany. #Initials of the primary contributor(s) responsible for acquiring the PGC-LC-MS/MS data, see Chatterjee et al., 2019 for details [1]. [*]Indicates non-cancer cell line.

Supplementary Table 2: Details of cultured cells investigated in this study

Cancer type	Cell lines (origin/ identifier)	Age (years)	Sex	Source	Tumourigenic
**1. Brain cancer**					
A. Glioblastoma	i. U87MG[a] (ATCC HTB-14)	-	M	Glioblastoma	Yes
	ii. A172 (ATCC CRL-1620)	53	M	Glioblastoma	No
	iii. U118MG[a] (CLS 300362)	50	M	Glioblastoma	Yes
	iv. U138MG[a] (CLS 300363)	47	M	Glioblastoma	Yes
B. Neuroblastoma	i. SK-N-B(2)[b] (ATCC CRL-2271)	2	M	Neuroblastoma	Yes
**2. Blood cancer**					
A. APL	i. HL-60 (ATCC CCL-240)	36	F	Promyelocytes from peripheral blood	Yes
B. AML	i. THP1 (ATCC TIB-202)	1	M	Monocytes from peripheral blood	No
C. ALL	i. CCRF-CEM (ATCC CCL-119)	4	F	T-lymphoblasts from peripheral blood	Yes
**3. Melanoma skin cancer**	i. MM253 (CBA-1347)	-	-	Lymph node	Yes
**4. BC**	i. HMEC[*] (ATCC PCS600-010	-	F	Cuboidal epithelium BC	No
	ii. HS578T (ATCC HTB-126)	74	F	Sarcoma (Basal B)	No
	iii. MCF7 (ATCC HTB-22)	69	F	Pleural effusion, luminal adeno-carcinoma	No
	iv. MDA-MB157 (ATCC HTB-24)	44	F	Pleural effusion, luminal adeno-carcinoma	Yes
	v. MDA-MB231 (ATCC HTB-26)	51	F	Pleural effusion, metastatic adeno-carcinoma (Basal B)	Yes
	vi. MDA-MB468 (ATCC HTB-132)	51	F	Pleural effusion, Luminal adeno-carcinoma	Yes
	vii. SKBR3 (ATCC HTB-30)	43	F	Pleural effusion, HER2-enriched adeno-carcinoma	Yes
**5. Lung cancer**	i. A549 (ATCC CCL-185)	58	M	Lung tissues	Yes
**6. HCC**	i. HepG2 (ATCC HB-8065)	15	M	HCC tissues	Yes
**7. CRC**	i. SW480 (ATCC CCL-228)	50	M	Dukes' Type B CRC (rectum)	Yes
	ii. SW620 (ATCC CCL-227)	51	M	Dukes' Type C CRC (Colon)	Yes
	iii. SW837 (ATCC CCL-235)	53	M	Dukes' Type C CRC (Rectum)	Yes
	iv. SW1116 (ATCC CCL-233)	73	M	Dukes' Type A CRC (Large Intestine, Colon)	Yes
	v. LIM1215[c]	34	M	Hereditary nonpolyposis CRC, Ileocecal valve, Omental metastasis	Yes
	vi. LIM1899[c]	-	-	Columnar cell carcinoma of invasive colon, moderately differentiated	Yes
	vii. LIM2405[c]	-	M	Columnar cell carcinoma of invasive colon, poorly differentiated	Yes

**8. OvC**	i. A2780 (ATCC CRL-2772)	-	F	Serous (ovary)	Yes
	ii. IGROV1 (ATCC TCP-1021)	47	F	Endometroid and serous (right ovary)	Yes
	iii. OVCAR3 (ATCC HTB-161)	60	F	Serous (ovary, ascites fluid)	Yes
	iv. SKOV3 (ATCC HTB-77)	64	F	Serous (ovary, ascites fluid)	Yes
	v. HOSE 6.3[*d]	-	F	Normal ovary	No
	vi. HOSE 17.1[*d]	-	F	Normal ovary (HOSE 6.3 and 17.1 are infected with human papillomavirus type 16)	No
**9. BlaCa**	i. J82 (ATCC HTB-1)	58	M	Transitional cell carcinoma (basal)	Yes
	ii. RT112 (ATCC TCP-1020)	-	F	Transitional cell carcinoma (luminal)	Yes
	iii. UROtsa[*e]	12	F	"Normal" urothelium immortalized using SV40 large T-antigen	No

[*]Indicates non-cancer cell line. [a]Gift from Professor Torsten Pietsch, Institute of Neuropathology, University of Bonn Medical Center, Bonn, Germany. [b]Gift from Prof Maria Kavallaris, Children's Cancer Institute, Lowy Cancer Research Centre, University of New South Wales, Sydney, NSW, Australia. [c]Ludwig Institute for Cancer Research, Parkville, VIC, Australia. [d]Garvan Institute of Medical Research, Sydney, NSW, Australia. [e]Gift from Professor Philip Erben, Department of Urology, Medical Faculty Mannheim, Heidelberg University, Mannheim, Germany.

Supplementary Table 3: Overview of the tissue samples investigated in this study and relevant ethics details

Cancer type	Primary contributor#	Biological replicates (*n*)	Sample type	Ethics details	Ref
**1. Blood cancer**					
A. CLL	KS	i. 8	FF	Project #8935 University of Sydney, Sydney, Australia	-
**2. Non-melanoma skin cancer**					
A. BCC<br>B. SCC	UM	i. 14<br>ii. 20<br>i. 15	FF BCC (P)<br>FFPE BCC (P)<br>FFPE SCC (P)	127-11-18042011 University of Leipzig, Leipzig, Germany	[2]
**3. GC**	VV	i. 3	FF (P)	Comitato Etico Regione Toscana (Tuscany, Italy)	-
**4. HCC**	HH	i. 3	FFPE (P)	Klinieka Bolnica Merkur (Zagreb, Croatia)	[8]
**5. CRC**	JHLC	i. 6	FF (P)	X08-0614 Sydney South West Area Health Service, Australia	[4]
	MKS	ii. 5	FF (P)	5201100040 Yonsei University, Seoul, South Korea	[5]
**6. PCa**	RKS	i. 50 tumour<br>5 BPH	FF	nº2695126 Faculdade de Medicina do Estado de São Paulo, Brazil	[24]

#Initials of the primary contributor responsible for acquiring the PGC-LC-MS/MS data, see Chatterjee et al. 2019 for details [1].

Supplementary Table 4: Details of individual tissue samples investigated in this study

Cancer type	Patient #	Age (years)	Sex	Localization	Tumour invasion level[$]	Diff. status/ doubling time
**1. Blood cancer**						
**A. CLL**	FF 1	–	–	Blood	Stable	> 5 years
	FF 2	–	–	Blood	Intermediate	< 18 months
	FF 3	–	–	Blood	Intermediate	< 18 months
	FF 4	–	–	Blood	Intermediate	< 18 months
	FF 5	–	–	Blood	Intermediate	< 18 months
	FF 6	–	–	Blood	Progressive	< 6 months
	FF 7	–	–	Blood	Progressive	< 6 months
	FF 8	–	–	Blood	Progressive	< 6 months
**2. Non-melanoma skin cancer**						
**A. BCC**	FF 1	74	M	Lower Leg	IV	–
	FF 2	79	M	Shoulder	IV	–
	FF 3	79	M	Shoulder	IV	–
	FF 4	74	M	Lower leg	IV	–
	FF 5	73	M	Back	IV	–
	FF 6	72	M	Chest	IV	–
	FF 7	82	M	Shoulder	IV	–
	FF 8	90	M	Back	III	–
	FF 9	88	M	Shoulder	IV	–
	FF 10	80	F	Cheek	IV	–
	FF 11	70	M	Chest	IV	–
	FF 12	82	M	Shoulder	IV	–
	FF 13	75	M	Shoulder	IV	–
	FF 14	80	M	Shoulder	IV	–
	FFPE 1	71	M	Nose	IV	–
	FFPE 2	25	F	Ear	III	–
	FFPE 3	88	M	Nose	IV	–
	FFPE 4	25	F	Head	III	–
	FFPE 5	79	M	Back	III	–
	FFPE 6	79	M	Neck	IV	–
	FFPE 7	72	F	Cheek	IV	–
	FFPE 8	75	F	Nose	IV	–
	FFPE 9	90	M	Chest	IV	–
	FFPE 10	62	M	Nose	IV	–
	FFPE 11	73	F	Nose	IV	–
	FFPE 12	82	F	Nose	IV	–
	FFPE 13	72	M	Cheek	IV	–
	FFPE 14	62	M	Shoulder	IV	–
	FFPE 15	77	F	Forehead	IV	–
	FFPE 16	80	F	Chin	IV	–
	FFPE 17	84	M	Lower leg	IV	–
	FFPE 18	81	M	Cheek	IV	–
	FFPE 19	77	M	Cheek	IV	–
	FFPE 20	58	M	Nose	V	–
**B. SCC**	FFPE 1	65	F	Cheek	IV	–
	FFPE 2	85	M	Head	V	–
	FFPE 3	72	F	Head	IV	–
	FFPE 4	90	M	Head	V	–
	FFPE 5	72	M	Nose	V	–
	FFPE 6	74	F	Cheek	IV	–
	FFPE 7	73	F	Upper trunk	III	–
	FFPE 8	76	M	Cheek	IV	–
	FFPE 9	74	M	Head	IV	–
	FFPE 10	79	F	Head	IV	–
	FFPE 11	90	M	Head	V	–
	FFPE 12	88	M	Ear	IV	–
	FFPE 13	64	M	Head	IV	–
	FFPE 14	87	M	Ear	V	–
	FFPE 15	78	M	Head	V	–
**3. GC**	FF 1	70	M	Gastric	G3	
	FF 2	64	M	Gastric	NG	
	FF 3	76	F	Gastric	G2	
**4. HCC**	–	–	–	–	–	–
**5. CRC**	FF 1	76	F	Rectum	B1/T3N0M0	Moderate
	FF 2	84	F	Colon	B1/T3N0M0	Moderate
	FF 3	56	F	Rectum	B1/T3N0M0	Moderate
	FF 4	57	M	–	B1/T3N0M0	Moderate
	FF 5	74	F	–	B1/T3N0M0	Moderate
	FF 6	–	–	–	–	–

	FF 1	64	M	Sigmoid, polyploid	I	Well
	FF 2	69	M	Rectum, ulcerative	IIIB	Moderate
	FF 3	67	M	Cecum, ulcero fungating	IIIB	Moderate
	FF 4	40	M	Rectum, annular constrictive	IV	Well
	FF 5	73	M	Transverse, ulcero fungating	IV	Poor
**6. PCa**	FF 1	62	M	Prostate	3/3/1 (T1c)	–
	FF 2	68	M	Prostate	3/3/1 (T3a)	–
	FF 3	54	M	Prostate	3/3/1 (T2b)	–
	FF 4	60	M	Prostate	3/3/1 (T1c)	–
	FF 5	52	M	Prostate	3/3/1 (T1c)	–
	FF 6	52	M	Prostate	3/3/1 (T1c)	–
	FF 7	59	M	Prostate	3/3/1 (T1c)	–
	FF 8	59	M	Prostate	3/3/1 (T2c)	–
	FF 9	64	M	Prostate	3/3/1 (T2a)	–
	FF 10	67	M	Prostate	3/3/1 (T1c)	–
	FF 11	67	M	Prostate	3/4/2 (T2b)	–
	FF 12	72	M	Prostate	3/4/2 (T1c)	–
	FF 13	50	M	Prostate	3/4/2 (T1c)	–
	FF 14	72	M	Prostate	3/4/2 (T2b)	–
	FF 15	63	M	Prostate	3/4/2 (T1c)	–
	FF 16	68	M	Prostate	3/4/2 (T1c)	–
	FF 17	58	M	Prostate	3/4/2 (T1c)	–
	FF 18	68	M	Prostate	3/4/2 (T1c)	–
	FF 19	66	M	Prostate	3/4/2 (T1c)	–
	FF 20	57	M	Prostate	3/4/2 (T1c)	–
	FF 21	79	M	Prostate	4/3/3 (T1c)	–
	FF 22	55	M	Prostate	4/3/3 (T1c)	–
	FF 23	70	M	Prostate	4/3/3 (T2c)	–
	FF 24	64	M	Prostate	4/3/3 (T1c)	–
	FF 25	66	M	Prostate	4/3/3 (T1c)	–
	FF 26	62	M	Prostate	4/3/3 (T2b)	–
	FF 27	70	M	Prostate	4/3/3 (T1c)	–
	FF 28	62	M	Prostate	4/3/3 (T1c)	–
	FF 29	55	M	Prostate	4/3/3 (T2a)	–
	FF 30	48	M	Prostate	4/3/3 (T2a)	–
	FF 31	71	M	Prostate	4/4/4 (T2b)	–
	FF 32	69	M	Prostate	4/4/4 (T2b)	–
	FF 33	56	M	Prostate	4/4/4 (T2c)	–
	FF 34	50	M	Prostate	4/4/4 (T2c)	–
	FF 35	66	M	Prostate	4/4/4 (T2c)	–
	FF 36	75	M	Prostate	4/4/4 (T2b)	–
	FF 37	60	M	Prostate	4/4/4 (T2b)	–
	FF 38	66	M	Prostate	4/4/4 (T1c)	–
	FF 39	63	M	Prostate	4/4/4 (T1c)	–
	FF 40	63	M	Prostate	4/4/4 (T1c)	–
	FF 41	60	M	Prostate	5/5/5 (T2c)	–
	FF 42	68	M	Prostate	5/5/5 (T2b)	–
	FF 43	56	M	Prostate	5/5/5 (T2c)	–
	FF 44	59	M	Prostate	5/5/5 (T2c)	–
	FF 45	66	M	Prostate	5/5/5 (T2c)	–
	FF 46	65	M	Prostate	5/5/5 (T2a)	–
	FF 47	70	M	Prostate	5/5/5 (T3c)	–
	FF 48	73	M	Prostate	5/5/5 (T2c)	–
	FF 49	58	M	Prostate	5/5/5 (T1c)	–
	FF 50	65	M	Prostate	5/5/5 (T1c)	–
	FF 51	3.5	M	Prostate	BPH	–
	FF 52	10.2	M	Prostate	BPH	–
	FF 53	6.9	M	Prostate	BPH	–
	FF 54	4.4	M	Prostate	BPH	–
	FF 55	2.3	M	Prostate	BPH	–

[$]Please note that the annotations of the tumour grade and invasion level, which were performed by trained pathologists across multiple institutions, vary between the investigated cancer types and subtypes.

Supplementary Table 5: Details of unpaired tumour/non-tumour tissue samples (spotted in technical duplicates) from commercial FFPE-TMA slides investigated by MALDI-MSI in this study

Organ	Diagnosis	Age (years)	Sex	Tumour history	Differentiation/tumour grade
Skin	Tumour	43	M	N/A	N/A
	Non-tumour	N/A	F	–	–
Colorectal	Tumour	54	M	7 months	Moderate/T3N0M0
	Non-tumour	24	M	–	–
Liver	Tumour	65	M	15 days	Moderate/ T1N0M0
	Non-tumour	30	M	–	–
Gastric	Tumour	48	M	1 month	Poor / T3N1M0
	Non-tumour	66	M	–	–

Supplementary Table 6: Distribution (relative abundance) of the *N*-glycan types across various protein fractions (WCL, MF, S) extracted from the studied human cancer cell lines.

Cancer type	Cell lines_Sample type	Distribution of *N*-glycan types			
		Paucimannose	Oligomannose	Oligomannose-related structure	Complex/ hybrid
**1. Brain cancer**					
A. Glioblastoma	i. U87MG_MF	6.87%	32.50%	2.69%	57.94%
	U87MG _WCL	14.34%	46.27%	1.44%	37.96%
	ii. A172_MF	13.25%	55.02%	3.34%	28.39%
	A172_S	1.20%	7.91%	0.28%	90.61%
	A172_WCL	29.97%	46.01%	3.69%	20.34%
	iii. U118MG_MF	6.82%	35.93%	2.15%	55.10%
	U118MG_WCL	4.92%	19.39%	1.34%	74.35%
	iv. U138MG_MF	7.51%	57.88%	3.85%	30.76%
	U138MG _WCL	8.21%	25.29%	0.83%	65.67%
B. Neuroblastoma	i. SK-N-B(2)	1.80%	58.84%	1.07%	38.29%
**2. Blood cancer**					
A. APL	i. HL60_WCL				
	Differentiated	19.91%	74.06%	3.45%	2.58%
	Undifferentiated	2.68%	84.25%	6.96%	6.11%
B. AML	i. THP1_MF				
	Infected	11.76%	35.37%	2.24%	50.63%
	Uninfected	11.82%	37.88%	1.31%	48.99%
	THP1_WCL				
	Infected	40.22%	35.96%	1.75%	23.57%
	Uninfected	50.20%	34.31%	1.90%	12.09%
C. ALL	i. CCRF-CEM_MF	0.00%	32.81%	2.12%	65.70%
**3. Melanoma skin cancer**	i. MM253	6.74%	65.94%	2.66%	24.66%
**4. BC**	i. HMEC*_MF	0.00%	62.93%	1.45%	35.62%
	HMEC*_S	0.00%	6.11%	0.00%	93.89%
	ii. HS578T_MF	0.00%	51.39%	0.00%	48.61%
	HS578T_S	0.00%	5.28%	0.00%	94.72%
	iii. MCF7_MF	3.28%	76.76%	4.11%	15.84%
	MCF7_S	0.89%	23.53%	0.29%	75.29%
	iv. MDA-MDA-MB157_MF	11.59%	47.93%	2.35%	38.13%
	MDA-MB157_S	3.21%	12.90%	0.00%	83.89%
	v. MDA_MB231_ MF	0.29%	41.46%	3.20%	55.05%
	MDA-MB231_S	9.49%	11.41%	0.00%	79.10%
	vi. MDA_MB468_ MF	11.33%	77.14%	3.11%	8.41%
	MDA-MB468_S	2.07%	10.39%	0.00%	87.54%
	vii. SKBR3_MF	6.18%	49.73%	2.82%	41.27%
	SKBR3_S	1.03%	15.48%	0.00%	83.49%
**5. Lung cancer**	i. A549_MF	5.68%	69.90%	1.39%	23.03%
	A549_WCL	5.01%	70.78%	1.49%	22.72%
**6. HCC**	i. HepG2_MF	6.69%	55.16%	3.94%	34.21%
	HepG2_WCL	18.85%	62.00%	5.35%	13.79%
**7. CRC**	i. SW480_MF	1.38%	55.82%	0.63%	42.17%
	ii. SW620_MF	2.77%	50.18%	0.27%	46.78%
	iii. SW837_MF	1.55%	59.97%	0.44%	38.05%
	iv. SW1116_MF	1.52%	49.78%	0.73%	47.97%
	v. LIM1215_MF	16.74%	72.31%	0.58%	10.37%
	vi. LIM1899_MF	19.62%	68.36%	1.35%	11.68%
	vii. LIM2405_MF	20.31%	54.98%	3.96%	20.75%
**8. OvC**	i. A2780_MF	10.20%	56.18%	1.16%	32.47%
	ii. IGROV1_MF	3.69%	60.40%	1.38%	34.53%
	iii. OVCAR3_MF	3.56%	61.38%	1.72%	33.35%
	iv. SKOV3_MF	1.96%	57.62%	1.10%	39.32%
	v. HOSE 6.3*_MF	1.49%	20.96%	0.32%	77.22%
	vi. HOSE 17.1*_MF	2.64%	37.36%	0.57%	59.43%
**9. BlaCa**	i. J82_MF	2.86%	40.63%	0.90%	55.62%
	J82_WCL	2.50%	40.22%	1.05%	56.23%
	ii. RT112_MF	1.95%	45.28%	1.17%	51.60%
	RT112_WCL	1.98%	55.59%	1.86%	40.57%
	iii. UROtsa*_MF	3.02%	61.39%	1.50%	34.09%
	UROtsa*_ WCL	2.33%	48.28%	0.96%	48.43%

*indicates non-cancer cell line

Supplementary Table 7: Distribution (relative abundance) of the individual oligomannosidic species across various protein fractions (WCL, MF, S) extracted from the studied human cancer cell lines

Cancer type	Cell lines_Sample type	Distribution of oligomannose				
		M5	M6	M7	M8	M9
**1. Brain cancer**						
A. Glioblastoma	i. U87MG_MF	2.26%	5.25%	5.92%	9.57%	9.50%
	U87MG _WCL	5.56%	11.22%	10.14%	11.15%	8.19%
	ii. A172_MF	1.87%	6.47%	16.01%	18.62%	12.05%
	A172_S	2.45%	0.98%	1.66%	2.01%	0.81%
	A172_WCL	3.42%	6.37%	10.61%	13.80%	11.81%
	iii. U118MG_MF	1.20%	4.74%	5.70%	13.17%	11.12%
	U118MG_WCL	1.26%	3.02%	2.64%	6.15%	6.32%
	iv. U138MG_MF	2.12%	7.09%	9.28%	20.22%	19.16%
	U138MG _WCL	1.72%	3.93%	3.95%	7.59%	8.10%
B. Neuroblastoma	i. SK-N-B(2)	3.02%	4.57%	4.92%	11.52%	34.81%
**2. Blood cancer**						
A. APL	i. HL60_WCL					
	Differentiated	5.83%	13.39%	23.35%	22.60%	18.80%
	Undifferentiated	2.52%	12.76%	12.86%	32.85%	23.25%
B. AML	i. THP1_MF					
	Infected	5.49%	4.80%	6.42%	8.95%	12.21%
	Uninfected	4.89%	3.91%	8.61%	6.84%	13.69%
	THP1_WCL					
	Infected	3.50%	3.38%	7.02%	9.09%	11.32%
	Uninfected	3.82%	3.95%	9.71%	7.64%	13.31%
C. ALL	i. CCRF-CEM_MF	0.12%	0.00%	5.26%	11.23%	15.57%
**3. Melanoma skin cancer**	i. MM253	1.79%	11.24%	18.74%	16.63%	17.54%
**4. BC**	i. HMEC*_MF	0.00%	8.23%	11.12%	22.66%	20.91%
	HMEC*_S	0.61%	1.97%	3.52%	0.00%	0.00%
	ii. HS578T_MF	0.00%	6.56%	4.58%	18.77%	21.47%
	HS578T_S	0.00%	1.23%	1.43%	2.21%	0.41%
	iii. MCF7_MF	2.98%	10.29%	10.65%	28.40%	24.45%
	MCF7_S	4.26%	5.20%	6.08%	4.94%	3.06%
	iv. MDA-MB157_MF	3.30%	6.90%	9.66%	13.73%	14.34%
	MDA-MB157_S	1.40%	3.27%	3.19%	3.55%	1.49%
	v. MDA_MB231_MF	0.36%	2.82%	4.69%	7.78%	25.81%
	MDA-MB231_S	1.01%	2.10%	3.45%	3.43%	1.43%
	vi. MDA_MB468_MF	7.41%	9.77%	7.68%	22.27%	30.02%
	MDA-MB468_S	1.85%	2.18%	2.70%	2.04%	1.61%
	vii. SKBR3_MF	3.00%	9.42%	9.03%	16.13%	12.16%
	SKBR3_S	0.91%	3.61%	4.12%	4.20%	2.64%
**5. Lung cancer**	i. A549_MF	1.00%	19.27%	17.00%	20.08%	12.55%
	A549_WCL	1.02%	19.16%	18.05%	21.09%	11.46%
**6. HCC**	i. HepG2_MF	3.58%	7.46%	6.77%	18.39%	18.96%
	HepG2_WCL	9.06%	8.44%	9.82%	19.48%	15.20%
**7. CRC**	i. SW480_MF	1.46%	3.07%	9.29%	2.11%	39.89%
	ii. SW620_MF	4.63%	6.22%	12.47%	24.51%	2.35%
	iii. SW837_MF	0.61%	7.07%	14.63%	17.56%	20.12%
	iv. SW1116_MF	0.86%	5.09%	10.57%	15.43%	17.82%
	v. LIM1215_MF	2.93%	22.99%	45.67%	0.25%	0.46%
	vi. LIM1899_MF	1.17%	4.19%	8.18%	16.01%	37.81%
	vii. LIM2405_MF	2.98%	18.98%	31.57%	0.55%	0.90%
**8. OvC**	i. A2780_MF	1.59%	4.78%	12.17%	17.07%	20.57%
	ii. IGROV1_MF	0.60%	7.64%	13.01%	15.48%	23.68%
	iii. OVCAR3_MF	2.14%	4.75%	11.18%	18.86%	24.44%
	iv. SKOV3_MF	0.69%	4.62%	8.81%	18.85%	24.65%
	v. HOSE 6.3*_MF	0.29%	1.85%	4.63%	7.40%	6.78%
	vi. HOSE 17.1*_MF	0.63%	2.85%	6.13%	13.82%	13.92%
**9. BlaCa**	i. J82_MF	3.89%	8.65%	7.27%	15.06%	5.75%
	J82_WCL	3.38%	9.45%	6.26%	15.71%	5.43%
	ii. RT112_MF	2.16%	12.37%	8.93%	15.02%	6.80%
	RT112_WCL	2.85%	17.84%	10.16%	14.69%	10.06%
	iii. UROtsa*_MF	3.50%	17.83%	11.50%	14.90%	13.66%
	UROtsa*_WCL	2.68%	12.79%	9.59%	15.66%	7.55%

*Indicates non-cancer cell line

Supplementary Table 8: Distribution (relative abundance) of the *N*-glycan types across various sample types (FF, FFPE) extracted from the studied human cancer tumour vs non-tumour tissues

Cancer type	Sample type_Biological replicates	Distribution of *N*-glycan types			
		Paucimannose	Oligo-mannose	Oligomannose-related structure	Complex/hybrid
**1. Blood cancer**					
A. CLL	i. FF_Tumour Early stage_8	2.13%	64.49%	1.09%	32.30%
	FF_Tumour Late stage_8	3.37%	60.79%	1.03%	34.81%
**2. Non-melanoma skin cancer**					
A. BCC	i. FF_Non tumour_14	0.88%	4.84%	0.37%	93.91%
	FF_Tumour_14	1.53%	8.98%	0.59%	88.90%
	ii. FFPE_Non tumour_20	3.25%	8.30%	0.55%	87.90%
	FFPE_Tumour_20	3.85%	10.21%	0.45%	85.49%
B. SCC	i. FFPE_Non tumour_15	2.66%	8.68%	0.00%	88.66%
	FFPE_Tumour_15	3.89%	13.10%	0.00%	83.01%
**3. GC**	i. FF_Non tumour_3	1.36%	23.96%	0.00%	74.68%
	FF_Tumour_3	3.35%	21.62%	0.00%	75.03%
**4. HCC**	i. FFPE_Non tumour_3	4.78%	29.03%	1.28%	64.91%
	FFPE_Tumour_3	29.00%	25.91%	1.60%	43.49%
**5. CRC**	i. FF_Non tumour_6	1.24%	26.42%	0.85%	71.49%
	FF_Tumour_6	2.72%	37.79%	0.55%	57.96%
	ii. FF_Non tumour_5	3.52%	28.33%	1.35%	66.80%
	FF_Tumour_5	5.65%	37.18%	1.71%	55.46%
**6. PCa**	i. FF_Non tumour_5	3.06%	11.92%	0.66%	84.36%
	FF_Tumour_Stage1_10	5.12%	11.18%	1.08%	82.62%
	FF_Tumour_Stage2_10	5.48%	11.49%	0.77%	82.26%
	FF_Tumour_Stage3_10	6.53%	10.69%	1.02%	81.76%
	FF_Tumour_Stage4_10	6.68%	10.85%	0.76%	81.71%
	FF_Tumour_Stage5_10	4.50%	9.55%	0.63%	85.32%

Supplementary Table 9: Distribution (relative abundance) of the individual oligomannosidic species across various sample types (FF, FFPE) extracted from the studied human cancer tumour vs non-tumour tissues

Cancer type	Sample type_Biological replicates	Distribution of *N*-glycan types				
		M5	M6	M7	M8	M9
**1. Blood cancer**						
A. CLL	i. FF_Tumour Early stage_8	1.41%	8.05%	11.91%	18.26%	24.87%
	FF_Tumour Late stage_8	1.86%	6.72%	12.42%	21.38%	18.43%
**2. Non-melanoma skin cancer**						
A. BCC	i. FF_Non tumour_14	1.17%	1.27%	0.79%	0.92%	0.69%
	FF_Tumour_14	1.63%	2.28%	1.61%	2.02%	1.44%
	ii. FFPE_Non tumour_20	1.22%	1.76%	1.78%	2.09%	1.45%
	FFPE_Tumour_20	1.33%	2.18%	1.41%	0.78%	1.78%
B. SCC	i. FFPE_Non tumour_15	1.97%	1.75%	1.41%	1.89%	1.67%
	FFPE_Tumour_15	2.10%	2.44%	2.14%	3.49%	2.93%
**3. GC**	i. FF_Non tumour_3	4.89%	2.80%	1.67%	5.84%	8.67%
	FF_Tumour_3	3.99%	3.89%	3.29%	5.06%	4.99%
**4. HCC**	i. FFPE_Non tumour_3	2.13%	7.56%	9.73%	6.73%	2.89%
	FFPE_Tumour_3	7.12%	7.16%	7.32%	2.73%	1.59%
**5. CRC**	i. FF_Non tumour_6	3.14%	2.90%	4.04%	6.04%	10.36%
	FF_Tumour_6	4.13%	3.08%	6.54%	8.93%	15.11%
	ii. FF_Non tumour_5	5.38%	4.31%	4.29%	5.67%	8.69%
	FF_Tumour_5	7.43%	7.81%	5.69%	7.08%	9.17%
**6. PCa**	i. FF_Non tumour_5	5.91%	3.18%	1.19%	1.17%	0.46%
	FF_Tumour_Stage1_10	5.50%	3.14%	1.30%	1.05%	0.19%
	FF_Tumour_Stage2_10	5.80%	2.99%	1.24%	0.98%	0.48%
	FF_Tumour_Stage3_10	5.36%	3.01%	1.14%	0.95%	0.22%
	FF_Tumour_Stage4_10	5.10%	3.00%	1.21%	1.08%	0.46%
	FF_Tumour_Stage5_10	4.49%	2.67%	1.14%	0.92%	0.34%
